# Obesity and Sex-Related Associations With Differential Effects of Sucralose vs Sucrose on Appetite and Reward Processing: A Randomized Crossover Trial

**DOI:** 10.1001/jamanetworkopen.2021.26313

**Published:** 2021-09-28

**Authors:** Alexandra G. Yunker, Jasmin M. Alves, Shan Luo, Brendan Angelo, Alexis DeFendis, Trevor A. Pickering, John R. Monterosso, Kathleen A. Page

**Affiliations:** 1 Division of Endocrinology, Department of Medicine, Keck School of Medicine, University of Southern California, Los Angeles; 2 Diabetes and Obesity Research Institute, Keck School of Medicine, University of Southern California, Los Angeles; 3 Department of Psychology, University of Southern California, Los Angeles; 4 Department of Preventive Medicine, Keck School of Medicine, University of Southern California, Los Angeles

## Abstract

**Importance:**

Nonnutritive sweeteners (NNSs) are used as an alternative to nutritive sweeteners to quench desire for sweets while reducing caloric intake. However, studies have shown mixed results concerning the effects of NNSs on appetite, and the associations between sex and obesity with reward and appetitive responses to NNS compared with nutritive sugar are unknown.

**Objective:**

To examine neural reactivity to different types of high-calorie food cues (ie, sweet and savory), metabolic responses, and eating behavior following consumption of sucralose (NNS) vs sucrose (nutritive sugar) among healthy young adults.

**Design, Setting, and Participants:**

In a randomized, within-participant, crossover trial including 3 separate visits, participants underwent a functional magnetic resonance imaging task measuring blood oxygen level–dependent signal in response to visual cues. For each study visit, participants arrived at the Dornsife Cognitive Neuroimaging Center of University of Southern California at approximately 8:00 am after a 12-hour overnight fast. Blood was sampled at baseline and 10, 35, and 120 minutes after participants received a drink containing sucrose, sucralose, or water to measure plasma glucose, insulin, glucagon-like peptide_(7-36)_, acyl-ghrelin, total peptide YY, and leptin. Participants were then presented with an ad libitum meal. Participants were right-handed, nonsmokers, weight-stable for at least 3 months before the study visits, nondieters, not taking medication, and with no history of eating disorders, illicit drug use, or medical diagnoses. Data analysis was performed from March 2020 to March 2021.

**Interventions:**

Participants ingested 300-mL drinks containing either sucrose (75 g), sucralose (individually sweetness matched), or water (as a control).

**Main Outcomes and Measures:**

Primary outcomes of interest were the effects of body mass index (BMI) status and sex on blood oxygen level–dependent signal to high-calorie food cues, endocrine, and feeding responses following sucralose vs sucrose consumption. Secondary outcomes included neural, endocrine, and feeding responses following sucrose vs water and sucralose vs water (control) consumption, and cue-induced appetite ratings following sucralose vs sucrose (and vs water).

**Results:**

A total of 76 participants were randomized, but 2 dropped out, leaving 74 adults (43 women [58%]; mean [SD] age, 23.40 [3.96] years; BMI range, 19.18-40.27) who completed the study. In this crossover design, 73 participants each received water (drink 1) and sucrose (drink 2), and 72 participants received water (drink 1), sucrose (drink 2), and sucralose (drink 3). Sucrose vs sucralose was associated with greater production of circulating glucose, insulin, and glucagon-like peptide–1 and suppression of acyl-ghrelin, but no differences were found for peptide YY or leptin. BMI status by drink interactions were observed in the medial frontal cortex (MFC; *P* for interaction < .001) and orbitofrontal cortex (OFC; *P* for interaction = .002). Individuals with obesity (MFC, β, 0.60; 95% CI, 0.38 to 0.83; *P* < .001; OFC, β, 0.27; 95% CI, 0.11 to 0.43; *P* = .002), but not those with overweight (MFC, β, 0.02; 95% CI, –0.19 to 0.23; *P* = .87; OFC, β, –0.06; 95% CI, –0.21 to 0.09; *P* = .41) or healthy weight (MFC, β, –0.13; 95% CI, –0.34 to 0.07; *P* = .21; OFC, β, –0.08; 95% CI, –0.23 to 0.06; *P* = .16), exhibited greater responsivity in the MFC and OFC to savory food cues after sucralose vs sucrose. Sex by drink interactions were observed in the MFC (*P* for interaction = .03) and OFC (*P* for interaction = .03) after consumption of sucralose vs sucrose. Female participants had greater MFC and OFC responses to food cues (MFC high-calorie vs low-calorie cues, β, 0.21; 95% CI, 0.05 to 0.37; *P* = .01; MFC sweet vs nonfood cues, β, 0.22; 95% CI, 0.02 to 0.42; *P* = .03; OFC food vs nonfood cues, β, 0.12; 95% CI, 0.02 to 0.22; *P* = .03; and OFC sweet vs nonfood cues, β, 0.15; 95% CI, 0.03 to 0.27; *P* = .01), but male participants’ responses did not differ (MFC high-calorie vs low-calorie cues, β, 0.01; 95% CI, –0.19 to 0.21; *P* = .90; MFC sweet vs nonfood cues, β, −0.04; 95% CI, –0.26 to 0.18; *P* = .69; OFC food vs nonfood cues, β, −0.08; 95% CI, –0.24 to 0.08; *P* = .32; OFC sweet vs nonfood cues, β, –0.11; 95% CI, –0.31 to 0.09; *P* = .31). A sex by drink interaction on total calories consumed during the buffet meal was observed (*P* for interaction = .03). Female participants consumed greater total calories (β, 1.73; 95% CI, 0.38 to 3.08; *P* = .01), whereas caloric intake did not differ in male participants (β, 0.68; 95% CI, –0.99 to 2.35; *P* = .42) after sucralose vs sucrose ingestion.

**Conclusions and Relevance:**

These findings suggest that female individuals and those with obesity may be particularly sensitive to disparate neural responsivity elicited by sucralose compared with sucrose consumption.

**Trial Registration:**

ClinicalTrials.gov Identifier: NCT02945475

## Key Points

**Question:**

Are adiposity and sex associated with neural, metabolic, and behavioral responses to consumption of nonnutritive sweeteners (NNSs) vs nutritive sugar?

**Findings:**

In this randomized crossover trial, both obesity and female sex were associated with differential neural food cue responsivity in reward processing areas following ingestion of sucralose (an NNS) compared with sucrose (nutritive sugar).

**Meaning:**

These findings suggest that female individuals and those with obesity have greater neural reward responses to NNS vs nutritive sugar consumption, highlighting the need to consider individual biological factors that might influence the efficacy of NNS.

## Teaser

This randomized crossover trial examines neural reactivity to different types of high-calorie food cues, metabolic responses, and eating behavior following sucralose vs sucrose consumption among healthy young adults.

## Introduction

Nonnutritive sweeteners (NNSs) are increasingly consumed as an alternative to nutritive sweeteners as a way to satisfy the desire for sweet taste while providing few or no calories. Although NNSs are now used by more than 40% of US adults,^1^ the health consequences of NNS consumption are still highly debated. Overall, the existing literature shows mixed results on the effects of NNS on appetite, glucose metabolism, and body weight,^2,3,4,5,6^ with no clear consensus on whether NNSs are beneficial or harmful for health.^7,8^

Prior work^9,10,11,12,13,14^ provides evidence that brain areas involved in regulation of taste, reward, and homeostasis may respond differently to NNSs compared with nutritive sugars, yet a number of questions still remain. Of note, the majority of previous studies in humans examining brain responses to NNS compared with nutritive sweeteners have been largely limited to studies of individuals with normal weight^9,10,11,12,15,16,17^ and exclusively male cohorts.^10,11,12,15,16,18^ Prior studies have shown that appetitive responses to food cues are greater in individuals with obesity and in female participants,^19,20^ and exposure to NNS compared with nutritive sugar caused increases in energy intake and weight gain in female rats with diet-induced obesity, but not in female rats receiving a standard chow diet,^21^ suggesting that obesity and sex might influence the behavioral and metabolic consequences of NNS ingestion. We aimed to address important gaps in knowledge by using neuroimaging coupled with blood sampling and assessments of eating behavior to provide novel insights into how adiposity and sex are associated with the neurobehavioral and metabolic outcomes of acute NNS compared with nutritive sweetener ingestion. On the basis of evidence from prior neuroimaging and behavioral studies,^9,10,11,12,13,14,19,20,22,23,24,25,26,27,28,29^ we hypothesized that the acute consumption of drinks containing sucralose compared with sucrose would provoke differential neural, endocrine, and appetitive responses, and that these differences would differ by obesity and sex.

## Methods

### Study Overview

Data are from the Brain Response to Sugar study, an investigation of neuroendocrine responses to high-reward foods, and findings presented in this study are the primary results from the randomized crossover trial. Participants provided written informed consent compliant with the University of Southern California institutional review board, which approved the study. This study follows the Consolidated Standards of Reporting Trials (CONSORT) reporting guidelines. The trial protocol can be found in Supplement 1 and in an online digital repository.^30^

**Figure 1.**
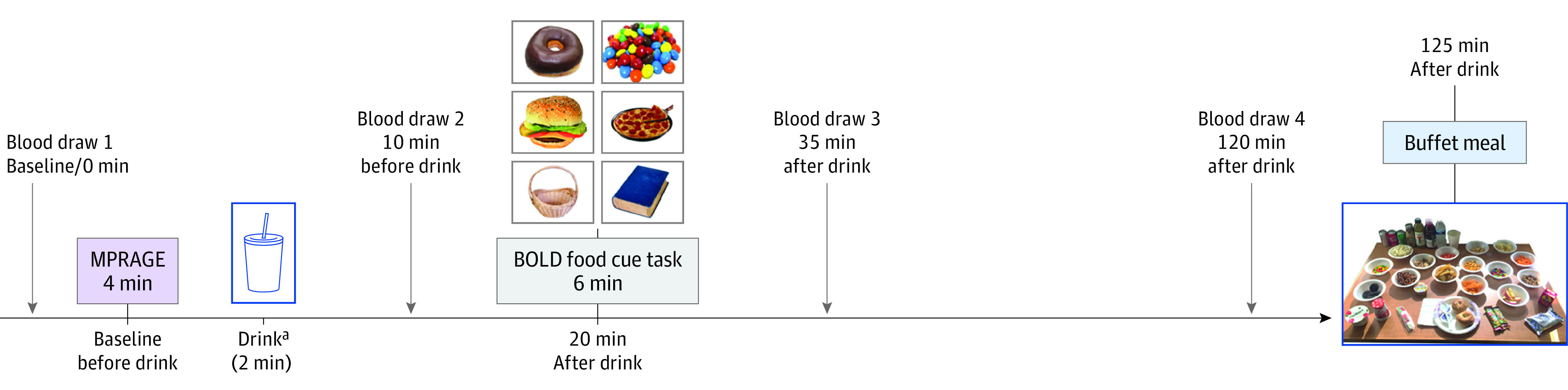
Overview of Study Visits BOLD indicates blood oxygen level–dependent; MPRAGE, 3D magnetization prepared rapid gradient echo sequence. ^a^Drinks were either 75 g of sucrose in 300 mL of water, sucralose (1.5, 2, or 3 mM based on individual sweetness match to sucrose drink) in 300 mL of water, or plain water (300 mL).

The Brain Response to Sugar within-participant randomized crossover trial included 4 drink conditions: glucose, sucralose, sucrose, and water. The data analyzed for the present article included sucralose, sucrose, and water to test the a priori hypothesis that the NNS, sucralose, would have differential effects on appetite and reward processing compared with the nutritive sugar, sucrose. The sucrose and sucralose drinks were individually sweetness matched during the initial screening visit (see eAppendix 1 in Supplement 2 for details). The water control drink was used to better interpret the directionality of differences. Glucose was included in the larger trial for the purposes of testing differences in equicaloric sugars (ie, glucose vs sucrose) on outcomes.^29^ Thus, the data reported here include a screening visit and 3 magnetic resonance imaging (MRI) study visits. For each study visit, participants arrived at the Dornsife Cognitive Neuroimaging Center of University of Southern California at approximately 8:00 am after a 12-hour overnight fast. The MRI visits were performed in blinded, random order (using function randperm, a computer-generated randomization procedure in MATLAB software version 2013b [MathWorks]) on separate days, and the interval between each study visit ranged from 2 days to 2 months. Participants ingested drinks containing either sucrose, sucralose, or a water control (see eAppendix 1 in Supplement 2 for details) and then underwent a food cue task (described later) beginning at approximately 20 minutes after ingesting the drink. Blood samples were collected at baseline (0 minutes), 10 minutes, 35 minutes, and 120 minutes after the drink, and the study ended with a food buffet at 125 minutes after the drink. For additional detailed study overview, see eAppendix 1 in Supplement 2 and Figure 1.

### Participants

Participants were aged 18 to 35 years, right-handed, nonsmokers, weight-stable for at least 3 months before the study visits, nondieters, not taking medication (except oral contraceptives), and with no history of diabetes, eating disorders, illicit drug use, or other medical diagnoses. Recruitment occurred between July 2016 and March 2020. We originally estimated a sample size of 120 participants to detect a minimum effect of 0.31 SD of the difference in sweeteners on activation within brain regions of interest (ROIs), controlling for the false discovery rate among brain regions, assuming a paired 2-sided *t* test, α = .05, and 80% power. The study was halted on March 13, 2020, because of the COVID-19 pandemic, with a recruited sample of 76 participants. We calculated that, with this sample, we would have 59% power to detect the original effect size of 0.31 SD, and using the same assumptions, we would have 80% power to detect a minimum effect size of 0.40 SD.

### Food Cue Task

Participants completed the food cue task in the MRI scanner by viewing stimuli through a mirror mounted over the head coil. In a randomized block design, participants were presented with a total of 12 blocks using MATLAB software version 2013b (MathWorks) and Psychtoolbox version 3.0.11 (MathWorks) on a 13-inch, 2.5-GHz Intel Core i5 processor MacBook Pro (Apple). There were different food cue types presented: within the food image blocks, there was a subset of 4 high-calorie and 4 low-calorie image blocks. In addition, high-calorie food cues were further subcategorized as 2 sweet and 2 savory image blocks (eTable 1 in Supplement 2). As a contrast, 4 nonfood image blocks were used (eTable 1 in Supplement 2). The set of food and nonfood cue images was matched for visual appeal and gathered from the food-pics database^31^ and prior published work^32^; for a full list and description of visual cue types and block categories see eAppendix 1 and eTable 1 in Supplement 2. Four images per block were presented in random order, each appearing immediately after the last. Within a block, each image was presented for 4 seconds. An 8-second questioning period followed each block where participants were asked to rate their hunger, wanting, and liking for the visual cues by clicking on a number (range, 1-5, where 1 denotes not at all, and 5 denotes very much) using a bimanual fiber optic response device. The total running time of this task was approximately 6 minutes.

### MRI Parameters and Analysis

Data were collected using a 3-T Magnetom Prismafit MRI System (Siemens Healthineers) with a 32-channel head coil. A high-resolution 3D magnetization prepared rapid gradient-echo sequence (repetition time, 1950 milliseconds; echo time, 2.26 milliseconds; bandwidth, 200Hz/pixel; flip angle, 9°; slice thickness, 1 mm; field of view, 224 × 256 mm; matrix, 224 × 256) was used to acquire structural images for multiparticipant registration. The blood oxygen level–dependent (BOLD) response was measured with a multiband interleaved gradient-echo planar imaging sequence to identify relative activation in brain ROIs using the contrasts of food (high-calorie plus low-calorie) vs nonfood cues, high-calorie (sweet plus savory) vs nonfood cues, high-calorie vs low-calorie food cues, sweet vs nonfood cues, and savory vs nonfood cues to examine BOLD responses to specific types of food cues. Eighty-eight 1.5-mm-thick slices covering the whole brain were acquired using the following parameters: repetition time, 1000 milliseconds; echo time, 43.20 milliseconds; bandwidth, 2055 Hz/pixel; flip angle, 52°; field of view, 128 × 112 mm; and matrix, 128 × 112. A priori brain ROIs included 8 brain regions implicated in feeding regulation: the nucleus accumbens, amygdala, dorsal striatum, medial frontal cortex (MFC), hippocampus, insula, orbitofrontal cortex (OFC), and hypothalamus^33,34,35,36^ (see eFigure 1 in Supplement 2 for anatomical template of ROI). All ROIs were bilateral and anatomically defined using the Harvard-Oxford Cortical and Subcortical Atlas found in the FMRIB Software Library version 6.0 (FMRIB Analysis Group) using a voxel probability threshold greater than 50%, except the hypothalamus, which is not included in the atlas and was defined bilaterally as a 2-mm spherical ROI surrounding peak glucose-responsive voxels identified previously.^35^ The percentage of BOLD signal change was extracted from each ROI and cue contrast for each participant to identify differences in relative brain activation to food cues vs nonfood cues using FSL’s FEATquery. For additional details on MRI analysis, see eAppendix 1 in Supplement 2.

### Metabolite and Hormone Analysis

Plasma glucose was measured enzymatically using glucose oxidase (YSI 2300 STAT PLUS Enzymatic Electrode-YSI analyzer, Yellow Springs Instruments). Plasma insulin, glucagon-like peptide–1 (GLP-1_(7-36)_) (active), acyl-ghrelin (active ghrelin), and leptin were measured via Luminex multiplex technology (Millipore), and peptide YY (PYY) (total) was measured using a human PYY enzyme-linked immunosorbent assay kit (Millipore). To represent the overall response to ingestion of each drink, we calculated total area under the curve (AUC) using the trapezoid method across the 120-minute testing period.^37,38,39^

### Ad Libitum Buffet Meal

Study sessions ended with the presentation of an ad libitum buffet meal given 125 minutes after the drink. For a detailed overview of the ad libitum buffet meal assessment, see eAppendix 1 and eTable 2 in Supplement 2. To give an index of the degree of compensation for the 300 kcal sucrose preload during the ad libitum buffet meal, we calculated percentage compensation index scores^40^ (see eAppendix 1 in Supplement 2).

### Habitual NNS and Dietary Intake Assessment

We administered repeated 24-hour dietary recalls at the screening visit and each study visit to determine the proportion of participants who consume NNS in their diet. See eAppendix 1 in Supplement 2 for details.

### Statistical Analysis

We evaluated the effect of sucralose vs sucrose ingestion on the following primary outcomes: (1) percentage BOLD signal change to high-calorie vs nonfood food cue contrasts; (2) circulating glucose, insulin, GLP-1, PYY, acyl-ghrelin, and leptin levels; and (3) ad libitum feeding responses after consumption of sucralose compared with sucrose. Secondary outcomes included neural, endocrine, and feeding responses following the sucrose vs water (control) and sucralose vs water (control) comparisons and in-scanner cue-induced appetite (hunger, liking, and wanting) ratings after each visual block following sucralose vs sucrose (and vs water as a control), and those results are reported in eAppendix 2 in Supplement 2. We used several linear mixed-effects regression models to examine the effect of sucralose vs sucrose on the aforementioned outcomes. Given our a priori hypothesis regarding effects of body mass index (BMI; calculated as weight in kilograms divided by height in meters squared) status and sex on outcomes, we tested for 2-way interactions between (1) BMI status and drink condition and (2) sex and drink condition, where BMI status and sex were treated as categorical variables. For results that showed a significant BMI status by drink interaction or sex by drink interaction, in post hoc analyses, we stratified results by BMI status or sex to understand the stratum-specific effects. We also examined for 3-way interactions between BMI status, sex, and drink condition (sucralose vs sucrose) on percentage BOLD signal change to food vs nonfood cues as an exploratory post hoc analysis. A priori covariates included in the linear mixed-effects regression models were age,^41,42^ sex,^29,43^ BMI status,^29,44^ and NNS user status,^13,14^ with a random intercept for drink randomization order. For longitudinal models that included repeated measurements over time, a random intercept for participant was included with an unstructured covariance matrix. We treated each ROI independently, and all neural BOLD results were false discovery rate–corrected for the 8 ROI and 6 food cue contrast comparisons, in addition to adjusting for all covariates. By use of linear mixed-effects regression, *P* < .05 was interpreted as statistically significant. SAS statistical software version 9.4 (SAS Institute) was used for all data analyses. For additional detailed statistical methods, see eAppendix 1 in Supplement 2. Data analysis was performed from March 2020 to March 2021.

## Results

### Participants

**Figure 2.**
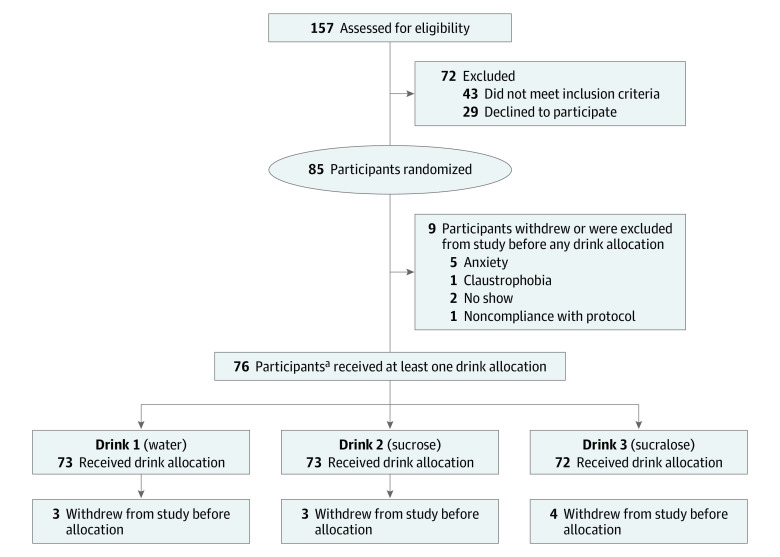
Participant Enrollment Flowchart for the Randomized Crossover Brain Response to Sugar II Trial ^a^Of the 76 participants who received at least 1 drink allocation, 2 participants received neither of the primary drink (ie, drink 2 or drink 3) allocations because of drop-out, and therefore were excluded from this analysis (n = 74).

A total of 76 participants were randomized, but 2 dropped out, leaving 74 adults (43 women [58%]; mean [SD] age, 23.40 [3.96] years; BMI range, 19.18-40.27) who completed the study. In this crossover design, 73 participants each received water (drink 1) and sucrose (drink 2), and 72 participants received water (drink 1), sucrose (drink 2), and sucralose (drink 3). Participants consumed a mean (SD) of 21.47 (51.70) mg per day of NNS in their habitual diet (44 NNS nonusers, 30 NNS users). NNS dietary use in our cohort was 41%, which is consistent with current National Health and Nutrition Examination data among US adults,^1^ and NNS user status did not differ by BMI (*F*_2,73_ = 0.42; *P* = .66) or sex (*t*_32_ = 0.25; *P* = .80) groups. Detailed participant characteristics are provided in Table 1 and Figure 2.

**Table 1.** Participant Characteristics

Characteristic	Participants, No. (%) (N = 74)
Age, mean (SD) [range], y	23.40 (3.96) [18.00-35.00]
Sex	
Male	31 (42)
Female	43 (58)
Body mass index, mean (SD) [range]^a^	27.22 (5.18) [19.18-40.27]
Healthy weight (≥18 to <25)	27 (37)
Overweight (≥25 to <30)	24 (32)
Obese (≥30)	23 (31)

^a^ Body mass index is calculated as weight in kilograms divided by height in meters squared.

### Neural BOLD Signal Response to Food Compared With Nonfood Cues After Sucralose vs Sucrose Drink

#### Whole Cohort

We did not observe significant differences in BOLD signal to any food cue contrasts in response to sucralose vs sucrose ingestion after adjusting for covariates, 8 ROIs, and 6 visual cue contrast comparisons (eTable 3 in Supplement 2). For secondary outcomes (ie, sucralose and sucrose vs water (control) comparisons), see eAppendix 2 and eTable 4 and eTable 5 in Supplement 2.

#### Effects of BMI Status

We observed BMI status by drink (sucralose vs sucrose) interactions in response to savory food vs nonfood cues in the MFC (*P* for interaction < .001) and OFC (*P* for interaction = .002), adjusted for covariates (age, sex, and NNS user status), multiple ROIs, and visual cue contrast comparisons. Similar interactions were observed in the MFC and OFC for the food vs nonfood and high-calorie vs nonfood contrasts, but these associations did not meet the threshold of significance (eTable 6, eTable 7, eTable 8, and eTable 9 in Supplement 2).

In data stratified by BMI status, we found that after sucralose vs sucrose ingestion, individuals with obesity had greater BOLD signal in response to savory vs nonfood cues in the MFC (β, 0.60; 95% CI, 0.38 to 0.83; *P* < .001), whereas participants with overweight (β, 0.02; 95% CI, −0.19 to 0.23; *P* = .87) or healthy weight (β, –0.13; 95% CI, –0.34 to 0.07; *P* = .21) did not have differential BOLD responses in the MFC (eFigure 2 in Supplement 2). Similar patterns were observed in the OFC, where individuals with obesity exhibited greater BOLD signal to savory food vs nonfood cues after sucralose compared with sucrose consumption (β, 0.27; 95% CI, 0.11 to 0.43; *P* = .002), and differences were not observed in overweight (β, –0.06; 95% CI, –0.21 to 0.09; *P* = .41) or healthy weight groups (β, –0.08; 95% CI, –0.23 to 0.06; *P* = .16) (eFigure 2 in Supplement 2).

#### Effects of Sex

There were sex by drink (sucralose vs sucrose) interactions in response to food vs nonfood cues in the MFC (*P* for interaction = .03), hippocampus (*P* for interaction = .03), and OFC (*P* for interaction = .03); in response to high-calorie vs low-calorie food cues in the dorsal striatum (*P* for interaction = .04), MFC (*P* for interaction = .04), insula (*P* for interaction = .04), and OFC (*P* for interaction = .04); in response to sweet vs nonfood cues in the MFC (*P* for interaction = .02) and OFC (*P* for interaction = .04); and after low-calorie vs nonfood in the MFC (*P* for interaction = .01), OFC (*P* for interaction = .01), hippocampus (*P* for interaction = .02), dorsal striatum (*P* for interaction = .03), and insula (*P* for interaction = .03), adjusted for covariates (age, BMI status, and NNS user status) and multiple ROI and visual cue contrast comparisons. The remaining associations did not meet the threshold of significance (eTable 10 in Supplement 2).

**Figure 3.**
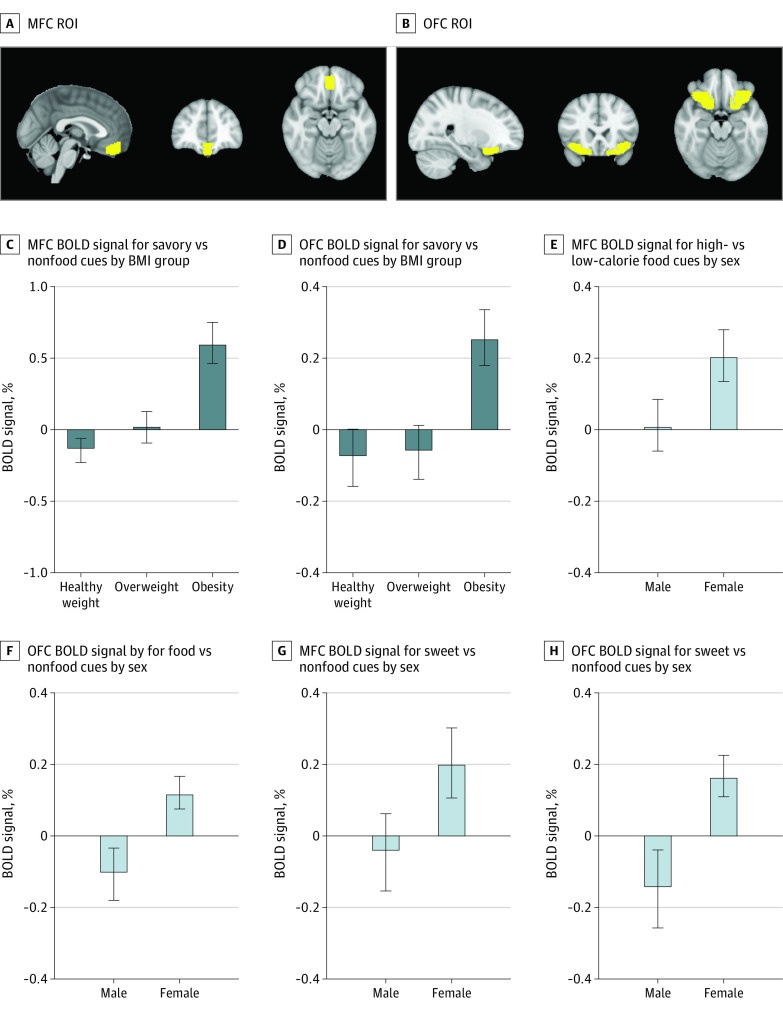
Brain Magnetic Resonance Images and Blood Oxygen Level–Dependent (BOLD) Signals Panels A and B show region of interest (ROI) masks for medial frontal cortex (MFC) (total voxels = 568; center voxel, 0 x-axis, 44 y-axis, −19 z-axis) and orbitofrontal cortex (OFC) (total voxels, 1695; center voxel left, −29 x-axis, 21 y-axis, and −17 z-axis; center voxel right, 28 x-axis, 22 y-axis, and −17 z-axis) derived from the Harvard-Oxford subcortical atlas. Data in panels C through H, show MFC and OFC BOLD signal after consumption of sucralose vs sucrose stratified by body mass index (BMI) group (C and D) and sex (E-H), in food cue contrasts where significant interactions between BMI group and drink or between sex and drink were found. Data are unadjusted mean and SEM (denoted by the error bars) for visual and interpretive purposes, but all statistical analyses were adjusted for covariates and multiple ROI and food cue contrast comparisons.

We found that female participants had greater BOLD response in the OFC to food vs nonfood cues after consuming sucralose vs sucrose (β, 0.12; 95% CI, 0.02 to 0.22; *P* = .03), whereas male participants did not have differential OFC responses to food vs nonfood cues between those 2 drink conditions (β, –0.08; 95% CI, –0.24 to 0.08; *P* = .32) (Figure 3 and eTable 11 in Supplement 2). Furthermore, MFC response to high-calorie vs low-calorie cues was greater after sucralose vs sucrose ingestion among female participants (β, 0.21; 95% CI, 0.05 to 0.37; *P* = .01) but not male participants (β, 0.01; 95% CI, –0.19 to 0.21; *P* = .90) (Figure 3 and eTable 11 in Supplement 2). Correspondingly, both MFC and OFC responses to sweet vs nonfood cues were greater after consuming sucralose compared with sucrose in female participants (β, 0.22; 95% CI, 0.02 to 0.42; *P* = .03; and β, 0.15; 95% CI, 0.03 to 0.27; *P* = .01, respectively) but not male participants (β, –0.04; 95% CI, –0.26 to 0.18; *P* = .69; and β, –0.11; 95% CI, –0.31 to 0.09; *P* = .31, respectively) (Figure 3; eTable 11 in Supplement 2). The remaining associations did not meet the threshold of significance (eTable 11 in Supplement 2).

### Post Hoc Results on Combined Effects of BMI Status and Sex on Neural BOLD Signal Response to Food vs Nonfood Cues After Sucralose vs Sucrose Drink

We found a significant 3-way interaction between BMI status, sex, and drink condition on the MFC BOLD response to savory vs nonfood cues (*P* for interaction = .02), adjusted for covariates and multiple ROI and visual cue contrast comparisons, but the remaining associations did not meet the threshold of significance (eTable 12, eTable 13, eTable 14, eTable 15, eTable 16, eTable 17, eTable 18, and eTable 19 in Supplement 2). To understand the directionality of this 3-way interaction, we ran an additional exploratory post hoc analysis and stratified results by the 6 BMI status and sex groups (eFigure 3 and eAppendix 2 in Supplement 2). We found that female participants with obesity were contributing disproportionately to the 3-way interaction among BMI status, sex, and drink condition on MFC BOLD response to savory vs nonfood cues (least square mean for MFC BOLD signal after sucralose, 0.47 [95% CI, 0.22 to 0.72]; least square mean after sucrose, –0.26 [95% CI, –0.50 to –0.03]; *P* < .001). Differences were not found in other subgroups (see eAppendix 2 in Supplement 2).

### Endocrine Responses After Sucralose vs Sucrose Drink for the Whole Cohort

There were no differences in baseline systemic levels of glucose, insulin, GLP-1, acyl-ghrelin, PYY, or leptin between the sucralose, sucrose, and water conditions (Table 2 shows baseline values for each study visit). AUCs for glucose, insulin, and GLP-1 were increased and acyl-ghrelin was suppressed after the sucrose compared with the sucralose condition. Table 2 shows AUC results for the drink comparisons, and eFigure 2 in Supplement 2 shows trajectories for each metabolite and hormone.

**Table 2.** Metabolic Responses to Sucralose, Sucrose, and Water (Control)

Hormone or metabolite^a^	Median (IQR)						*P* value^b^	*P* value^c^	*P* value^d^
	Sucralose		Sucrose		Water				
	Baseline	AUC	Baseline	AUC	Baseline	AUC			
Glucose, mg/dL	88.09 (83.73-93.95)	10 417.11 (9939.24-11 035.24)	87.92 (83.01-91.42)	12 863.51 (11 544.47-13 994.18)	88.60 (83.81-94.33)	10 492.04 (9993.23-10 962.56)	<.001^e^	<.001^e^	.94
Insulin, μIU/mL	18.50 (8.91-33.59)	1735.37 (911.53-3620.23)	15.25 (8.97-31.01)	5759.43 (4531.12-9011.55)	13.29 (8.35-31.68)	1353.55 (912.18-3811.86)	<.001^e^	<.001^e^	.61
GLP-1_(7-36)_, pg/mL	3.54 (1.20-7.66)	311.51 (144.00-743.15)	2.98 (1.20-6.64)	904.09 (493.20-1465.35)	3.62 (1.20-8.44)	210.70 (144.00-677.13)	<.001^e^	<.001^e^	.68
Acyl-ghrelin, pg/mL	108.21 (80.17-169.26)	14 752.28 (8726.45-20 119.05)	118.25 (64.01-171.99)	8835.50 (5739.83-12 600.18)	104.85 (70.59-161.70)	12 280.23 (89 77.91-17 636.13)	<.001^e^	<.001^e^	.07
PYY_(total)_, pg/mL	76.88 (57.46-97.37)	7071.05 (5414.40-10 038.13)	75.94 (46.17-94.78)	7755.38 (5461.23-10 202.87)	71.93 (54.04-91.54)	6837.65 (5136.32-8692.66)	.55	.11	.31
Leptin, pg/mL	6308.00 (3188.00-10 969.00)	72 1212.50 (381 170.00-1 173 580.00)	5723.00 (3285.00-8713.00)	646 475 (341 630.00-1 083 612.50)	6230.00 (3152.50-11 640.00)	718 155 (356 565.00-1 342 061.25)	.08	.02^e^	.62

Abbreviations: AUC, area under the curve; GLP-1, glucagon-like peptide–1; PYY, peptide YY.

SI conversion factors: To convert glucose to millimoles per liter, multiply by 0.0555; insulin to picomoles per liter, multiply by 6.945.

^a^ Data are plasma concentrations of metabolites and hormones after ingestion of 75 g of sucrose in 300 mL of water, sucralose (1.5, 2, or 3 mM based on individual sweetness match to sucrose drink) in 300 mL of water, or plain water (300 mL).

^b^ Data are for sucralose vs sucrose.

^c^ Data are for sucrose vs water.

^d^ Data are for sucralose vs water.

^e^ Indicates *P* values are statistically significant at *P* < .05, adjusted for age, sex, body mass index status, and nonnutritive sweetener user status.

There were no BMI status by drink interactions on AUC for plasma glucose (*P* for interaction = .92), insulin (*P* for interaction = .07), GLP-1 (*P* for interaction = .44), PYY (*P* for interaction = .84), or leptin (*P* for interaction = .09) (eFigure 4 in Supplement 2). Although we found a significant BMI status by drink interaction on AUC for acyl-ghrelin (*P* for interaction = .03), stratified analyses revealed that individuals with healthy weight (β, –8716.14; 95% CI, –11139.00 to –6293.65; *P* < .001), overweight (β, –6046.44; 95% CI, –8580.14 to –3512.74; *P* < .001), and obesity (β, –3466.44; 95% CI, −4824.76 to −2108.13; *P* < .001) all had significantly greater suppression of acyl-ghrelin after sucrose compared with sucralose ingestion (eFigure 4 in Supplement 2). In addition, we did not find sex by drink interactions on AUC for plasma glucose (*P* for interaction = .32), insulin (*P* for interaction = .08), GLP-1 (*P* for interaction = .71), acyl-ghrelin (*P* for interaction = .69), PYY (*P* for interaction = .34), or leptin (*P* for interaction = .27) (eFigure 5 in Supplement 2).

### Eating Behavior After Sucralose vs Sucrose Drink

Mean degree of caloric compensation for the sucrose preload (ie, adjustment in caloric intake based on caloric preload form sucrose drink, or compensation index) and mean (SD) total caloric intake after each drink condition for the whole cohort and stratified by BMI status and sex are provided in eAppendix 2 in Supplement 2. For secondary outcomes (ie, sucralose and sucrose vs water (control) comparisons), see eAppendix 2 in Supplement 2.

#### Whole Cohort and Effects of BMI Status

 Participants consumed greater total calories (β, 1.37; 95% CI, 0.31 to 2.43; *P* = .01) during the ad libitum buffet meal after the sucralose compared with the sucrose drink condition but did not fully compensate for the 300 kcal sucrose preload (see eAppendix 2 in Supplement 2). With regard to effects of BMI status, we did not find an interaction between BMI status and drink condition on total caloric intake (*P* for interaction = .14) during the ad libitum buffet meal.

#### Effects of Sex

We found an interaction between sex and drink condition on total calories consumed (*P* for interaction = .03) during the buffet meal. In post hoc results stratified by sex, after ingestion of sucralose compared with sucrose, female participants consumed greater total calories (β, 1.73; 95% CI, 0.38 to 3.08; *P* = .01), whereas total caloric intake did not differ for male participants (β, 0.68; 95% CI, –0.99 to 2.35; *P* = .42) (eFigure 6 in Supplement 2).

## Discussion

In this randomized crossover trial, we found BMI status by drink interactions for BOLD signal response to viewing savory food vs nonfood cues in the MFC and OFC, and post hoc stratified analyses indicated that individuals with obesity, but not with overweight or healthy weight, exhibited greater BOLD percentage signal change to savory vs nonfood cues after sucralose compared with sucrose ingestion (Figure 3). Of note, both the MFC and OFC are regions of the brain implicated in mediating conditioned motivation to eat^23,45^ and encoding reward value or valence of food cues,^23,46,47^ and obesity has been shown to be associated with greater food cue reactivity within these prefrontal reward-related areas.^22,48,49^

We also found robust sex by drink interactions for BOLD signal response to several food cue contrasts in the MFC, hippocampus, OFC, insula, and dorsal striatum, such that female participants, but not male participants, exhibited greater BOLD signal, particularly in the MFC and OFC, to several food cue contrasts after consuming sucralose compared with sucrose (Figure 3). Notably, a study^43^ that examined the effects of sex on neural activation to viewing highly palatable foods from a fasted compared with fed state demonstrated that women, but not men, had higher BOLD signal to high-calorie food cues in a fasted state and decreased BOLD signal in a fed state within neural areas involved in reward-seeking behavior,^43,50,51^ suggesting that female participants have greater differential neural responses based on changes in fuel status.^43^ In concert with the aforementioned study,^43^ our current findings demonstrate that young female participants may have greater sensitivity toward nutrient sensing, which we postulate may have evolved as a protective mechanism for reproduction. In post hoc exploratory analyses, we found a 3-way interaction between BMI status, sex, and drink condition on the MFC BOLD response to savory vs nonfood cues, suggesting that female participants with obesity may be particularly sensitive to the effects of NNS vs nutritive sweetener consumption on neural food cue reactivity (eFigure 2 in Supplement 2). These findings support a previous report^21^ in a rodent model showing that exposure to NNS compared with nutritive sugar caused substantial increases in energy intake, weight gain, and adiposity in female rats with diet-induced obesity, but not in female rats receiving a standard chow diet. Notably, Swithers et al^21^ speculated that their findings may translate to humans where the appetitive consequences of consuming NNS may be more pronounced in women with obesity. Longer-term randomized clinical trials are warranted to further elucidate mechanisms underlying the roles of sex and obesity on NNS effects.

Endocrine responses to sucralose vs sucrose did not differ by BMI status or sex, and the acute ingestion of sucralose did not stimulate an increase in circulating glucose, insulin, GLP-1, acyl-ghrelin, PYY, or leptin in any participants (eFigure 2, eFigure 4, and eFigure 5 in Supplement 2). These findings are in line with prior reports^52,53,54,55,56,57^ showing that sucralose consumed in a fasted state and in isolation has no effect on plasma metabolites or appetite-regulating hormones.

Although some reports have suggested that acute NNS consumption compared with sugar-sweetened beverage or water consumption is associated with increases in food intake,^58,59^ others have shown that NNSs may have little to no direct effect on subsequent energy intake.^12,60,61^ Although we found a sex by drink interaction for total calories consumed during the buffet meal, indicating that female participants, but not male participants, had greater caloric intake after the sucralose vs sucrose condition (eFigure 6 in Supplement 2), neither male participants nor female participants fully compensated for the sucrose drink condition caloric preload (300 kcal).

### Limitations

To capture the 2-hour plasma glucose, insulin, and GLP-1 levels after drink ingestion, the ad libitum buffet meal in our study sessions occurred approximately 125 minutes after the drink preload, which may have reduced the ability to detect differences in eating behavior outcomes between the drink conditions.^62^ As part of our parent study design, participants were given a 75-g sucrose load (containing 300 kcal) in accordance with a standard oral glucose or sucrose tolerance test. Although this standard dose of sucrose is clinically relevant and is known to cause an increase in peripheral glucose and appetite-regulating hormones,^29^ future investigators should consider potential dose-dependent effects when examining differential neuroendocrine responses to sucrose compared with NNS ingestion. Furthermore, the unique chemical structure of each type of NNS may elicit varying physiological responses^63^ and subsequently could have differential effects on neuroendocrine regulation of appetite and feeding behavior. Although the goal of the present study was to examine the effects of acute consumption of sucralose when ingested in isolation, a recent report^64^ demonstrated that short-term daily consumption of sucralose with, but not without, carbohydrates was associated with impairments in insulin sensitivity, which was, in turn, associated with decreases in neural responses to sucrose. Consequently, whether the observed obesity-related and sex-related associations with differential responses to acute sucralose in this study would be different if consumed in combination with carbohydrates remains to be seen and should be examined in future studies.

## Conclusions

Our findings indicate that female individuals and those with obesity, and especially female individuals with obesity, might be particularly sensitive to greater neural responsivity elicited by sucralose compared with sucrose consumption. This study highlights the need to consider individual biological factors in research studies and potentially in dietary recommendations regarding the use and efficacy of NNS for body weight management.

## Notes

Supplement 1.
Trial Protocol

Supplement 2. **eAppendix 1. **Supplemental Methods **eAppendix 2.** Supplemental Results of Secondary Outcomes and Additional Post-Hoc Results **eTable 1.** Full List of Visual Cues and Respective Categories Used During the BOLD Food Cue Task **eTable 2.** Foods and Drinks Available at the Ad Libitum Buffet Meal Along With the Caloric Content of Each **eTable 3.** BOLD Signal in Regions-of-Interest (ROI) to Food Cue Contrasts Following Sucralose vs Sucrose Ingestion in Whole Cohort **eTable 4.** BOLD Signal in Regions-of-Interest (ROI) to Food Cue Contrasts Following Sucrose vs Water Ingestion in Whole Cohort **eTable 5.** BOLD Signal in Regions-of-Interest (ROI) to Food Cue Contrasts Following Sucralose vs Water Ingestion in Whole Cohort **eTable 6.** BMI Status by Drink (Sucralose vs Sucrose) Interactions for BOLD Signal in Regions-of-Interest (ROI) to Food Cue Contrasts **eTable 7.** BMI Status by Drink (Sucrose vs Water) Interactions for BOLD Signal in Regions-of-Interest (ROI) to Food Cue Contrasts **eTable 8.** Post-Hoc Analysis for MFC BOLD Signal to High-Calorie vs Non-Food and Savory vs Non-Food Contrasts Following Sucrose vs Water Ingestion, Stratified by BMI Status **eTable 9. **BMI Status by Drink (Sucralose vs Water) Interactions for BOLD Signal in Regions-of-Interest (ROI) to Food Cue Contrasts **eTable 10.** Sex by Drink (Sucralose vs Sucrose) Interactions for BOLD Signal in Regions-of-Interest (ROI) to Food Cue Contrasts **eTable 11.** Post-Hoc Analysis for BOLD Signal to Food Cue Contrasts Following Sucralose vs Sucrose Ingestion, Stratified by Sex **eTable 12.** Sex Status by Drink (Sucrose vs Water) Interactions for BOLD Signal in Regions-of-Interest (ROI) to Food Cue Contrasts **eTable 13.** Post-Hoc Analysis for BOLD Signal to Food Cue Contrasts Following Sucrose vs Water Ingestion, Stratified by Sex **eTable 14. **Sex Status by Drink (Sucralose vs Water) Interactions for BOLD Signal in Regions-of-Interest (ROI) to Food Cue Contrasts **eTable 15.** Post-Hoc Analysis for BOLD Signal to Food Cue Contrasts Following Sucralose vs Water Ingestion, Stratified by Sex **eTable 16. **Post-Hoc Analysis for Differences in LSmeans Between Drink Comparisons for In-Scanner Cue-Induced Hunger, Wanting, and Liking Ratings in Whole Cohort **eTable 17. **Post-Hoc Analysis for Differences in LSmeans Between Drink Comparisons for In-Scanner Cue-Induced Hunger Ratings, Stratified by BMI Status **eTable 18. **Post-Hoc Analysis for Differences in LSmeans Between Drink Comparisons for In-Scanner Cue-Induced Hunger, Liking, and Wanting Ratings, Stratified by Sex **eTable 19.** Post-Hoc 3-Way Interaction Between BMI Status, Sex, and Drink Condition (Sucralose vs Sucrose) on BOLD Signal in Regions-of-Interest (ROI) to Food Cue Contrasts **eFigure 1.** Sagittal, Coronal, and Axial Images Depicting the Anatomically Defined Brain Regions of Interest (ROIs) **eFigure 2.** Trajectories for Plasma Glucose and Hormones After Sucralose, Sucrose, and Water Drinks, Among Whole Cohort **eFigure 3.** MFC BOLD Signal Response to Savory vs Non-Food Cues After Sucralose Compared to Sucrose Ingestion, Stratified by Both BMI Status and Sex **eFigure 4.** Trajectories for Plasma Glucose and Hormones After Sucralose, Sucrose, and Water Drinks, Stratified by Body Mass Index (BMI) Status **eFigure 5.** Trajectories for Plasma Glucose and Hormones After Sucralose, Sucrose, and Water Drinks, Stratified by Sex **eFigure 6. **Difference in Total Caloric Intake Following Sucralose vs Sucrose Preload in the Whole Cohort and Stratified by Males and Females
eReferences

Supplement 3.
Data Sharing Statement
