# Supplementary material for: Obesity and Sex-Related Associations With Differential Effects of Sucralose vs Sucrose on Appetite and Reward Processing: A Randomized Crossover Trial
Source: JAMA Netw Open. 2021 Sep 28;4(9):e2126313. doi: 10.1001/jamanetworkopen.2021.26313 (PMC8479585; doi:10.1001/jamanetworkopen.2021.26313)
Supplement: Supplement 1. — Trial Protocol [file jamanetwopen-e2126313-s001.pdf]

IRB PROTOCOL NUMBER: HS-09-00395
ClinicalTrials.gov Identifier: NCT02945475

TITLE: Neural Mechanisms for Appetitive Responses to High Reward Foods (BRS-2)

STUDY PHASE: Proof of Concept

PRINCIPAL INVESTIGATOR(S): Kathleen A. Page, MD
USC Division of Endocrinology
2250 Alcazar Street; CSC 209
Los Angeles, CA 90089

CO-INVESTIGATOR(S): Shan Luo, PhD, John Monterosso, PhD

SPONSOR: National Institutes of Health (NIH) National Institute of Diabetes and Digestive and Kidney Diseases R01DK102794 (PI: K.A.P)

PARTICIPANTS/LOCATONS:
Dana & David Dornsife Cognitive Neuroscience Imaging Center and Diabetes and Obesity Research Institute at the Keck School of Medicine, University of Southern California

1.0 OBJECTIVES AND PURPOSE

Aim 1: Determine effects of acute ingestion of drinks with sweet taste + nutrient (sucrose) vs. sweet taste without nutrient (sucralose) on brain, endocrine and food intake responses, and examine the role of obesity on differential responses.

Aim 2: Determine the brain, endocrine, and food intake responses to acute ingestion of drinks containing two types of sugar (sucrose vs glucose) that are matched for caloric load but processed differently by the body, and examine the role of obesity on differential responses.

Aim 3: Determine the effects of consuming drinks with a sweet taste (sucralose) compared to non-sweet control (water) on brain, endocrine and food intake responses.

Primary Outcomes: (1) % BOLD signal change to high-calorie vs non-food food cue contrasts; (2) change in cerebral blood flow; (3) circulating glucose, insulin, GLP-1, PYY, acyl-ghrelin, and leptin levels; (4) total energy intake from ad-libitum meal

Secondary outcomes: (1) In-scanner cue-induced appetite (hunger, liking, and wanting) ratings after each visual block; (2) out of scanner appetite ratings; (3) correlations between brain and hormone responses; (4) correlations between appetite and brain responses; (5) functional connectivity between regions involved in regulation of food intake; (6) % BOLD signal change to other visual food cue contrasts (high-calorie+low-calorie) vs nonfood cues, high-calorie vs low-calorie food cues, sweet vs non-food cues, and savory vs non-food cues to examine BOLD responses to specific types of food cues.

2.0 Background and Rationale for the Study

Obesity rates have risen dramatically over the last two decades, such that now more than half a billion people worldwide are obese and over 1.4 billion are overweight (1). Temporal patterns show a correlation between rising intake of added sugars and the global obesity epidemic (2-3). Findings by our group and others suggest the fructose component of added sugar-sweeteners may act on the brain to stimulate food intake (4-6). In lean adults, we showed the ingestion of fructose failed to cause the reduction in hypothalamic activity that occurred following glucose ingestion (4), produced smaller increases in circulating levels of the satiety hormones, insulin and GLP-1, and no reduction in hunger, providing a potential neuroendocrine mechanism explaining epidemiologic data showing association between fructose intake and obesity (7). Real-world sugar sweeteners generally contain a mixture of the monosaccharides, glucose and fructose. The proposed study will determine brain, hormone and appetitive responses to sucrose (50% glucose and 50% fructose) and compare to pure glucose. We will measure circulating levels of hormones involved in appetite regulation (including insulin, PYY, ghrelin, GLP-1) and determine the brain responses to each type of sugar, providing important insights into physiological and brain responses to sugar ingestion.

In addition, in light of growing efforts to reduce sugar consumption, a greater number of people are now consuming non-nutritive sweeteners (NNS), which are often recommended as a way to satisfy cravings for sweet taste without increasing caloric intake (8). However, epidemiologic studies (9,10) and feeding studies in animal models (11,12) suggest NNS may stimulate food intake and contribute to obesity. There are several mechanisms underlying the paradoxical positive association between NNS and weight gain. First, the absence of calories in NNS could fail to stimulate physiological responses that reduce food intake (8). Second, the pleasant sweet taste of NNS may stimulate hedonic hunger and reward-motivated feeding (13). Third, NNS might disrupt hormones involved in hunger and satiety causing an increase in food-seeking behavior (8). Recent reviews of the literature have concluded existing evidence is insufficient to refute or support any of these mechanisms, emphasizing the need for more research (13,14). We propose to study the effects acute ingestion of the NNS, sucralose, on the activation of brain appetite and reward regions, changes in appetite hormones, and food intake as well as how obesity impacts these responses.

Food intake is regulated by a complex interaction between central and peripheral signals that serve to maintain energy balance (15) and survival. The hypothalamus interacts with other brain regions and the periphery to ensure adequate energy supply by regulating the homeostatic drive to eat. Other brain regions, *e.g.,* striatum, orbitofrontal cortex (OFC), amygdala and insula, control motivation-reward systems associated with the hedonic drive (pursuit of pleasure and reward) to eat (16,17). Hedonic motivations can override homeostatic control mechanisms and promote food intake, especially in an environment of abundant highly palatable food (15-17). Cortical regions, including the prefrontal cortex, regulate impulse control and inhibit the motivation for rewarding stimuli like food and drugs (18). These cortical, homeostatic and hedonic feeding regions are tightly interconnected and form an integrated network that regulates feeding behavior. We found differences in cerebral blood flow (CBF) in the hypothalamus after glucose vs. fructose ingestion (4). We propose to combine CBF and a food-cue task to gain novel insights into differential sugar and NNS effects on resting-state CBF and cue-induced activation of homeostatic, hedonic and cortical brain regions. This work provides new insights into the effects of different sugars and NNS on brain networks that regulate feeding.

3.0 STUDY DESIGN

This is a randomized crossover study in 40 healthy-weight, 40 obese and 40 overweight adults to compare brain, hormone and appetitive responses to an acute ingestion of 300 ml drinks containing: (1) 75 g glucose; (2) 75 g sucrose; (3) sucralose (sweetness matched to sucrose); (4) water alone. The drink order given during the fMRI sessions will be randomized using a computer-generated sequence. Participants and investigators are blinded to study drink condition.

3.1 Recruitment

Participants in the greater Los Angeles area will be recruited by flyers and advertisements and social media outlets.

3.2 SELECTION AND WITHDRAWAL OF SUBJECTS

Healthy young adults with no history of diabetes or impaired fasting glucose will be invited to participate in this study. Males and females, ages 18-35 years old will be screened with a history and physical examination. Subjects will also be required to read and complete the MR Safety Questionnaire. Female subjects will undergo the studies during the follicular phase of their menstrual cycle, as self reported.

Inclusion criteria:

- Age between 18-35 years
- Stable weight for at least 3 months prior to participation
- Healthy-weight: BMI 18.5-24.9 kg/m^2
- Obese: BMI $\geq$30 kg/m^2
- Overweight: BMI 25-29.9 kg/m^2

Exclusion criteria:

- Current or prior history of tobacco use and/or substance abuse
- history of type 1 or type 2 diabetes mellitus
- history of medical or surgical event or disorder that might affect the study outcome
- history of eating disorder
- current use of medications (except oral contraceptives)
- Individuals will be excluded if they have any of the following: cardiac pacemaker, implanted cardiac defibrillator, carotid artery vascular clamp, neurostimulator, cochlear implant, metal fragments (including shrapnel) in the head, eyes, or skin,

vascular stent, ocular implant, penile implant, vascular filter for clots (including Greenfield, Umbrella, or Birds Nest filters).

- Individuals with a history of claustrophobia
- Pregnancy (females)
- Left handed
- Visual impairments that won't allow them to view the screen inside the scanner without glasses
- Smoker
- Vegetarian

3.3 Sample Size and Power

We estimated sample size based on practical and statistical considerations. For pairwise comparisons a sample size of 120 (40 healthy-weight, 40 obese and 40 overweight participants) allows us to detect an effect size of 0.25 SD for differential effects of sweeteners on activation within brain regions of interest, *e.g.*, hypothalamus, nucleus accumbens, assuming a two-sided t-test, α=0.05, and 80% power, and after correcting for multiple comparisons we can detect an effect size of 0.31 SD. We previously observed a within-subject effect size of 0.6 SD for difference in hypothalamic CBF in response to glucose *vs.* fructose ingestion (4), well above the projected 0.25 SD in our sample size estimation. For between group comparisons, this sample size allows us to detect an effect size of 0.65. Feasibility of observing the between-group effect size is supported by data from our preliminary studies comparing differences between obese and lean groups in the reactivity to food cues within the nucleus accumbens, a region of interest in the reward system. In 12 lean and 12 obese participants, we observed a difference in BOLD signal change after glucose vs. fructose between lean and obese groups in the nucleus accumbens equal to an effect size of 0.74. Additionally, Masduda et al (28) found obese vs. lean differences in the hypothalamic response to glucose that correspond to effect sizes well above the 0.6 effect size that we have the power to detect with our study design.

3.4 Consenting and Screening: Consenting and screening will take place in a quiet, private room at the Dornsife Center at USC or at a private screening room at the KSOM Diabetes and Obesity Research Institute, or at the participant's home.

3.5 Measurements and Assessments at Visit 1

Anthropometric measurements: Measurements will be taken in a private room. Height will be measured to the nearest 0.1 cm using a portable stadiometer and weight to the nearest 0.1 kg using a portable scale. Waist and hip circumference will be measured to the nearest 0.1 cm. Waist circumference will be measured at the midpoint between the iliac crest and lower costal margin in the midaxillary line immediately above the iliac crest. Hip circumference will be measured by positioning the measuring tape around the maximum circumference of the buttocks. Waist-hip-ratio (WHR) will be calculated by dividing waist circumference by hip circumference. Percent body fat will be measured by a digital scale, body fat analyzer (Tanita, Tanita Corp of America, Inc) which uses bioelectrical impendance analysis to estimate percent body fat.

Sweetness Matching: Participants will be asked to rate the sweetness of drinks that were sweetened with sucrose (25% weight per volume concentration) or sucralose (1.5 mM, 2 mM, or 3 mM) in order to match the sweetness intensity of the sucralose and sucrose drinks for each participant.

Dietary Assessments: Diet will be the baseline screening visit and each MRI visit using the multipass 24-hour dietary recall, which is a validated method that provides detailed information on food and beverages consumed over the previous 24-hour period. Participants will be asked to recall all food and drinks items that they ingested during the previous 24-hours. In order to account for potential daily variations in dietary intake, 24-hour recalls will be captured on both weekdays and weekend days. Dietary data will be collected and compiled using the Nutrient Data System (NDS-R).

Physical Activity will be assessed using the 3-day physical activity recall.

3.6 Questionnaires

Socio-demographic questionnaire: participants will fill out a questionnaire related to socio-demographic information. It includes gender, age, education level, work status, employment type, and mothly household income.

Behavioral Activation Scale/Behavioral Inhibition Scale: Individual differences in sensitivity to reward and punishment will be measured by a behavioral inhibition/behavioral activation scale (BIS/BAS) developed by Carver and White.

Delay discounting scale: Subjects will be presented with a fixed set of 27 hypothetical choices between smaller immediate rewards (ranging from $11 to $80) and larger delayed rewards (ranging in amount from $20 to $85 and in delay from 7 to 186 days).

Three Factor Eating Questionnaire: Participants are asked questions about three factors involved in eating behavior: hunger, disinhibition and cognitive restraint.

Diet History Questionnaire II: Participants will be aksed questions related to the types and amount of foods that they typically ate over the past year. This questionnaire can be completed either on paper or online (http://riskfactor.cancer.gov/dhq2). It is provided free of charge by the National Institutes of Health for the purpose of collecting dietary information for research purposes.

4. MRI Sessions

Each study participant will undergo five magnetic resonance imaging (MRI) sessions in random order on separate days $\geq$ 2 days apart with one of the following stimuli: ingestion of sucrose (75 g sucrose dissolved in water to a volume of 10 ounces) or glucose (75 g glucose dissolved in water to a volume of 10 ounces) or water (volume of 10 ounces) or sucralose dissolved in water to a volume of 10 ounces.

Enrolled participants will report to the Dana and David Dornsife Center at the University of Southern California on the morning of the study. Weight will be obtained while particiapnts are in light clothing and without shoes on the morning of each study visit using a standard portable scale. Subjects will be asked to maintain their typical diets and equivalent exercise patterns during their participation in the study. Females will be studied during the follicular phase of their menstrual cycle. Dietary and physical activity questionnaires will be taken from each subject participant prior to each study session. Subjects will be asked to eat a similar meal on the evening prior to each study session and they will be asked to refrain from eating or drinking anything except water after 10pm the evening prior to each study session.

4.1 Drinks and Sugar Dose

Participants will ingest 300mL drinks containing either sucrose (75g), glucose (75g), sucralose (Tate & Lyle, London, UK) (sweetness matched), or a water control, mixed with 0.45g of non-sweetened zero calorie cherry flavoring (Kraft Foods Kool-Aid® Unsweetened Cherry Drink Mix) for palatability. The sucrose and sucralose drinks will be individually sweetness matched during the initial screening visit using a blinded task where participants select the concentration of sucralose (1.5 mM, 2 mM, or 3 mM) that best matches a 25% weight per volume concentration of sucrose. The order of the drinks will be randomized using a computer-generated sequence. Drinks will be prepared by the study coordinator. The rest of the study team and the participant will be blinded to drink type. Drinks will be administered from the same type of cup with a straw. Participants will be instructed to consume drinks in less than two minutes.

4.2 MRI Procedures

The MRI protocol includes three 8-minute ASL sequences to determine regional CBF responses at baseline (before drink consumption) and twice after drink consumption (+2 minutes and +22 minutes after drink). The food-cue task includes sixteen visual activation task blocks (8 Food blocks, 8 Non-food control blocks) presented in a randomized block design. Each block consists of 4 photographs presented in a random order. Each photograph is presented for 4 seconds with 1-second blank period between photographs. No photograph is presented more than once. At the end of each block, VAS appear and participants click on a number (1 to 5) using a computer mouse-like device to rate their feelings of hunger and desire to eat. BOLD acquisitions are acquired during the food cue task. The MRI session will also include a T1 structural scan (for anatomical registration). Appetite scales and blood samples are acquired at baseline and at +10, +35 and +120 minute post drink time points.

4.3 MRI Imaging Parameters

Images will be collected using a 3T Siemens MAGNETOM Prismafit MRI System, with a 32-channel head coil. A high-resolution 3D magnetization prepared rapid gradient echo (MPRAGE) sequence (TR=1950ms; TE=2.26ms; bandwidth=200Hz/pixel; flip angle=9°; slice thickness=1mm; FOV=224mm×256mm; matrix=224×256) will be used to acquire structural images for multi-subject registration. The blood oxygen level-dependent (BOLD) response will be measured with a multi-band interleaved gradient echo planar imaging sequence to identify relative activation in brain regions of interest using the contrasts of food and non-food cues. Eighty-eight 1.5-mm thick slices covering the whole brain were acquired using the following parameters: TR=1,000ms; TE=43.20ms; bandwidth=2,055Hz/pixel; flip angle=52°; FOV=128mm×112mm, matrix=128×112. *A priori* brain regions-of-interest (ROI) will include 8 brain regions implicated in feeding regulation: the nucleus accumbens, amygdala, dorsal striatum, medial frontal cortex (MFC), hippocampus, insula, orbitofrontal cortex (OFC), and hypothalamus. ROIs will be anatomically defined using the Harvard-Oxford Cortical and Subcortical Atlas found in FSL using a voxel probability threshold above 50%, except the hypothalamus, which is not included in the atlas and was defined bilaterally as a 2-mm spherical ROI surrounding peak glucose-responsive voxels identified previously (4). Percent BOLD signal change will be extracted from each ROI and cue contrast for each participant to identify differences in relative brain activation to food cues vs non-food cues using FSL's FEATquery. Food images Task: The block design will consist of of a total of 36 randomized blocks. 12 visual activation task blocks **(**4 high calorie high sugar food blocks, 4 high calorie low sugar food blocks, 4 non-food control blocks**)** will be presented in a randomized block design under each condition. Each block will consist of 4 photographs presented in random order. Each photograph will be presented for 3 seconds. At the end of each block, visual analogue scales will appear and subjects will rate their hunger and desire for food by clicking on a number (1 to 5) using a computer mouse-like device to rate their feelings of hunger and desire to eat sweet and savory foods on a scale from 1 to 5 where 1 is not at all and 5 is very much. The ASL sequences will use the QUIPSS-II method (29). A proximal inversion with a control for off-resonance effects (PICORE) mode was employed to provide high labeling efficiency (30). The ASL sequences were acquired along with one M0 image with following parameters: field of view (FOV) = 192 mm, matrix = 64 x 64, bandwidth = 2232 Hz/Pixel, slice thickness = 5 mm, interslice spacing = 0 mm, Repetition time (TR) = 4000 ms, echo time (TE) = 30 ms, flip angle = 90°, TI1/TIs/TI2 = 700/1,800/1,800 ms, label duration = 1675 ms, slab thickness = 100mm, in-plane resolution = 3 × 3 mm^2. The timing of the inversion pulses (TI) were optimized to reduce intravascular signal intensity at 3 T (31). The duration of the ASLs acquisition is 8 min and 18 seconds.

4.4 Blood Samples

At each MRI session, blood will be collected at times 0 (baseline), +20, +35, +120 minutes after drink ingestion. Blood will be assayed for hormones that are known to be involved in the regulation of obesity and appetite. DNA will be isolated from the baseline blood sample to examine for loci known to be associated with obesity and body weight. An intravenous catheter will be placed so that blood can be drawn from the cathether at 4 time points. To minimize discomfort, our participants will be given the option of having Emla Cream applied to help numb the skin area prior to inserting a needle

for the intravenous line. If there are technical difficulties and an intravenous catheter cannot be placed, then single blood draws will be performed at 3 time points instead.

4.5 Buffet Meal

After the MRI Scan, participants will be offered a buffet-style meal consisting of snack foods (eg. potato chips, apple slices, yogurt, candy bar, bagels with cream cheese) and drinks. All items will be presented on white plates and in standardized quantities (e.g. 70 g potato chip serving, 100 g candy bar serving, etc.). Subjects will be allotted 30 minutes to eat as much or as little of the buffet items as they want. All food items will be weighed to the nearest 0.1 gram. Total energy intake will be computed as the difference between the pre- and post-weights of all items. At the end of the buffet meal participant will be administered another appetite scale.

4.6 Appetite Ratings:

Hunger, fullness, satisfaction, and prospective food intake will be assessed at baseline, +30 and +60 min after drink consumption. The out of scanner behavioral scales will allow us to determine effects of drink on appetitive ratings without the addition of visual food cues.

4.7 Hormone Assays: Assays will be performed in the USC metabolic core lab at the Diabetes and Obesity Research Institute. Glucose and lactate will be assayed using a YSI analyzer, which uses a membrane bound glucose oxidase and lactate oxidase technique, respectively. Insulin, GLP-1 (active), peptide YY (PYY) (total), leptin and ghrelin (active) will be measured using Luminex multiplex technology (Millipore, St. Charles, MO), which uses fluorescent-coded magnetic beads with a reported intra-assay precision of 2-7% and inter-assay precision of 6-10% for the assays that we will be performing**.** Insulin Resistance**:** Fasting plasma insulin and glucose obtained at the screening visit will be used to estimate insulin resistance using the homeostatic model of insulin resistance (HOMA-IR).

5.0 MRI Analysis

We will use the Oxford University Centre for Functional MRI of the Brain Software Library (FMRIB), and MRI data will be processed using the fMRI Expert Analysis Tool (FEAT) version 6.00. fMRI data will be preprocessed using motion correction, high-pass filtering (100s), and spatial smoothing with a Gaussian kernel of full width at half-maximum=5mm. Functional data will be first mapped to each participant's anatomical image and then registered into standard space [Montreal Neurological Institute (MNI)] using affine transformation with FMRIB's Linear Image Registration Tool to the avg152 T1 MNI template. Food and non-food blocks will be added to the general linear model (GLM) after convolution with a canonical hemodynamic response function. Temporal derivatives and temporal filtering were added, increasing statistical sensitivity. Motion confounds were generated using the tool "fsl_motion_outliers" to be used as no-interest regressors in the GLM. For each participant, visual cue contrast maps will be created on the first-level analysis. To determine effects of drink ingestion on brain responses to food cues, we will perform a whole brain analysis to identify brain areas with relative increases or decreases in BOLD reactivity to food vs. non-food cues after drink ingestion. Main effect group contrast maps will be performed by comparing brain responses to food vs. non-food cues after drink ingestion at a significance threshold set at $p<0.05$, two-sided, FWE whole-brain correction. Contrast maps will also be performed comparing drink conditions; sucralose to sucrose conditions; sucrose to glucose; and sucralose to water conditions ($p<0.05$, 2-sided, FWE whole-brain corrected). Group comparisons will also be made on each of the above contrast maps ($p<0.05$, 2-sided, FWE whole-brain corrected). *A priori* brain regions-of-interest (ROI) will include 8 brain regions implicated in feeding regulation: the nucleus accumbens, amygdala, dorsal striatum, medial frontal cortex (MFC), hippocampus, insula, orbitofrontal cortex (OFC), and hypothalamus. ROIs will be anatomically defined using the Harvard-Oxford Cortical and Subcortical Atlas found in FSL using a voxel probability threshold above 50%, except the hypothalamus, which is not included in the atlas and was defined bilaterally as a 2-mm spherical ROI surrounding peak glucose-responsive voxels identified previously (4).

Bayesian Inference for ASL (BASIL) toolbox (https://fsl.fmrib.ox.ac.uk/fsl/fslwiki/BASIL) will be used to analyze the perfusion data. ASL data will be motion corrected and then tagged and untagged images were subtracted to obtain perfusion-weighted images (32). ASL volumes will be registered to the individual participant's T_1-weighted high-resolution anatomical volume using an affine registration with 12 degrees of freedom. Mean CBF across the whole brain and specifically in each ROI will be extracted. We will use whole brain CBF maps to identify brain areas with relative increases or decreases in CBF following drink ingestion. Main effect group contrast maps will be performed by subtracting CBF after drink from before drink at a significance threshold set at $p<0.05$, two-sided, FWE whole-brain correction. Comparisons will also be between drinks and between groups. For ROI analysis, mean CBF across the whole brain and specifically in each ROI will be extracted. The same 8 ROIs used for BOLD fMRI analysis will be used for ASL analysis. Atlas ROIs will be inverse-transformed to individual native space and regional CBF will be extracted for each ROI and participant.

6.0 Statistical Analyses

Linear mixed-effects regression models will be used to examine the the differences in effects of drink condition on the outcomes. We will test for interactions between: (1) weight group (obese, overweight, lean) and drink condition and (2) sex and drink condition, where weight group and sex will be treated as categorical variables. Exploratory analysis for three-way interactions between BMI status, sex, and drink condition on % BOLD signal change to food vs non-food cues and to change in CBF will be considered. A priori covariates included in the linear mixed-effects regression models will include age, sex, BMI, and NNS user status, with a random intercept for drink randomization order. For longitudinal models that include repeated measurements over time, a random intercept for subject will be included with an unstructured covariance matrix. We will treat each ROI independently and all BOLD and CBF results will be FDR-corrected for multiple comparisons. P values <0.05 will be interpreted as statistically significant. SAS 9.4 statistical software (SAS Institute, Cary, NC USA) will be used for all data analyses.

7.0 DATA COLLECTION AND MONITORING

Confidentiality of Discussions: The privacy of the subjects wil be protected by having discussions regarding the study and confidential health matters in a private consultation room in Dornsife or Clinical Science Center at HSC. All personnel have been HIPPA trained.

Data management and monitoring: All electronic and digital files will be stored on a secure network and access to the network will be password protected and encrypted. Individual identifiable data or the key cited above will not be stored on moveable media devices unless encrypted. All paper files will be stored in a locked file cabinet in a locked office and access is limited to members of the study research team.

8.0 ETHICAL AND REGULATORY CONSIDERATIONS

All institutional and Federal regulations concerning the Informed Consent form will be fulfilled. The study will be conducted in adherence to ICH Good Clinical Practice.

9.0 REFERENCES

1. WHO. 2013. Obesity and overweight.
2. Malik, V.S., and Hu, F.B. 2012. Sweeteners and Risk of Obesity and Type 2 Diabetes: The Role of Sugar-Sweetened Beverages. *Curr Diab Rep*.

3. Malik, V.S., Popkin, B.M., Bray, G.A., Despres, J.P., and Hu, F.B. 2010. Sugar-sweetened beverages, obesity, type 2 diabetes mellitus, and cardiovascular disease risk. *Circulation* 121:1356-1364.

4. Page, K.A., Chan, O., Arora, J., Belfort-Deaguiar, R., Dzuira, J., Roehmholdt, B., Cline, G.W., Naik, S., Sinha, R., Constable, R.T., et al. 2013. Effects of fructose vs glucose on regional cerebral blood flow in brain regions involved with appetite and reward pathways. *JAMA* 309:63-70.

5. Cha, S.H., Wolfgang, M., Tokutake, Y., Chohnan, S., and Lane, M.D. 2008. Differential effects of central fructose and glucose on hypothalamic malonyl-CoA and food intake. *Proc Natl Acad Sci U S A* 105:16871-16875.

6. Miller, C.C., Martin, R.J., Whitney, M.L., and Edwards, G.L. 2002. Intracerebroventricular injection of fructose stimulates feeding in rats. *Nutr Neurosci* 5:359-362.

7. Bray, G.A., Nielsen, S.J., and Popkin, B.M. 2004. Consumption of high-fructose corn syrup in beverages may play a role in the epidemic of obesity. *Am J Clin Nutr* 79:537-543.

8. Pepino, M.Y., and Bourne, C. 2011. Non-nutritive sweeteners, energy balance, and glucose homeostasis. *Curr Opin Clin Nutr Metab Care* 14:391-395.

9. Fowler, S.P., Williams, K., Resendez, R.G., Hunt, K.J., Hazuda, H.P., and Stern, M.P. 2008. Fueling the obesity epidemic? Artificially sweetened beverage use and long-term weight gain. *Obesity (Silver Spring)* 16:1894-1900.

10. Stellman, S.D., and Garfinkel, L. 1986. Artificial sweetener use and one-year weight change among women. *Prev Med* 15:195-202.

11. Swithers, S.E., and Davidson, T.L. 2008. A role for sweet taste: calorie predictive relations in energy regulation by rats. *Behav Neurosci* 122:161-173.

12. Swithers, S.E., Sample, C.H., and Davidson, T.L. 2013. Adverse effects of high-intensity sweeteners on energy intake and weight control in male and obesity-prone female rats. *Behav Neurosci* 127:262-274.

13. Mattes, R.D., and Popkin, B.M. 2009. Nonnutritive sweetener consumption in humans: effects on appetite and food intake and their putative mechanisms. *Am J Clin Nutr* 89:1-14.

14. Gardner, C., Wylie-Rosett, J., Gidding, S.S., Steffen, L.M., Johnson, R.K., Reader, D., and Lichtenstein, A.H. 2012. Nonnutritive sweeteners: current use and health perspectives: a scientific statement from the American Heart Association and the American Diabetes Association. *Circulation* 126:509-519.

15. Schwartz, M.W., Woods, S.C., Porte, D., Jr., Seeley, R.J., and Baskin, D.G. 2000. Central nervous system control of food intake. *Nature* 404:661-671.

16. Shin, A.C., Zheng, H., and Berthoud, H.R. 2009. An expanded view of energy homeostasis: neural integration of metabolic, cognitive, and emotional drives to eat. *Physiol Behav* 97:572-580.

17. Volkow, N.D., Wang, G.J., and Baler, R.D. 2011. Reward, dopamine and the control of food intake: implications for obesity. *Trends Cogn Sci* 15:37-46.
