# Supplementary material for: Obesity and Sex-Related Associations With Differential Effects of Sucralose vs Sucrose on Appetite and Reward Processing: A Randomized Crossover Trial
Source: JAMA Netw Open. 2021 Sep 28;4(9):e2126313. doi: 10.1001/jamanetworkopen.2021.26313 (PMC8479585; doi:10.1001/jamanetworkopen.2021.26313)
Supplement: Supplement 2. — eAppendix 1. Supplemental Methods eAppendix 2. Supplemental Results of Secondary Outcomes and Additional Post-Hoc Results eTable 1. Full List of Visual Cues and Respective Categories Used During the BOLD Food Cue Task eTable 2. Foods and Drinks Available at the Ad Libitum Buffet Meal Along With the Caloric Content of Each eTable 3. BOLD Signal in Regions-of-Interest (ROI) to Food Cue Contrasts Following Sucralose vs Sucrose Ingestion in Whole Cohort eTable 4. BOLD Signal in Regions-of-Interest (ROI) to Food Cue Contrasts Following Sucrose vs Water Ingestion in Whole Cohort eTable 5. BOLD Signal in Regions-of-Interest (ROI) to Food Cue Contrasts Following Sucralose vs Water Ingestion in Whole Cohort eTable 6. BMI Status by Drink (Sucralose vs Sucrose) Interactions for BOLD Signal in Regions-of-Interest (ROI) to Food Cue Contrasts eTable 7. BMI Status by Drink (Sucrose vs Water) Interactions for BOLD Signal in Regions-of-Interest (ROI) to Food Cue Contrasts eTable 8. Post-Hoc Analysis for MFC BOLD Signal to High-Calorie vs Non-Food and Savory vs Non-Food Contrasts Following Sucrose vs Water Ingestion, Stratified by BMI Status eTable 9. BMI Status by Drink (Sucralose vs Water) Interactions for BOLD Signal in Regions-of-Interest (ROI) to Food Cue Contrasts eTable 10. Sex by Drink (Sucralose vs Sucrose) Interactions for BOLD Signal in Regions-of-Interest (ROI) to Food Cue Contrasts eTable 11. Post-Hoc Analysis for BOLD Signal to Food Cue Contrasts Following Sucralose vs Sucrose Ingestion, Stratified by Sex eTable 12. Sex Status by Drink (Sucrose vs Water) Interactions for BOLD Signal in Regions-of-Interest (ROI) to Food Cue Contrasts eTable 13. Post-Hoc Analysis for BOLD Signal to Food Cue Contrasts Following Sucrose vs Water Ingestion, Stratified by Sex eTable 14. Sex Status by Drink (Sucralose vs Water) Interactions for BOLD Signal in Regions-of-Interest (ROI) to Food Cue Contrasts eTable 15. Post-Hoc Analysis for BOLD Signal to Food Cue Contrasts Following Sucralose v [file jamanetwopen-e2126313-s002.pdf]

Supplemental Online Content

Yunker AG, Alves JM, Luo S, et al. Obesity and sex-related associations with differential effects of sucralose vs sucrose on appetite and reward processing: a randomized crossover trial. *JAMA Netw Open*. 2021;4(9):e2126313. doi:10.1001/jamanetworkopen.2021.26313

eAppendix 1. Supplemental Methods

eAppendix 2. Supplemental Results of Secondary Outcomes and Additional Post-Hoc Results

eTable 1. Full List of Visual Cues and Respective Categories Used During the BOLD Food Cue Task

eTable 2. Foods and Drinks Available at the Ad Libitum Buffet Meal Along With the Caloric Content of Each

eTable 3. BOLD Signal in Regions-of-Interest (ROI) to Food Cue Contrasts Following Sucralose vs Sucrose Ingestion in Whole Cohort

eTable 4. BOLD Signal in Regions-of-Interest (ROI) to Food Cue Contrasts Following Sucrose vs Water Ingestion in Whole Cohort

eTable 5. BOLD Signal in Regions-of-Interest (ROI) to Food Cue Contrasts Following Sucralose vs Water Ingestion in Whole Cohort

eTable 6. BMI Status by Drink (Sucralose vs Sucrose) Interactions for BOLD Signal in Regions-of-Interest (ROI) to Food Cue Contrasts

eTable 7. BMI Status by Drink (Sucrose vs Water) Interactions for BOLD Signal in Regions-of-Interest (ROI) to Food Cue Contrasts

eTable 8. Post-Hoc Analysis for MFC BOLD Signal to High-Calorie vs Non-Food and Savory vs Non-Food Contrasts Following Sucrose vs Water Ingestion, Stratified by BMI Status

eTable 9. BMI Status by Drink (Sucralose vs Water) Interactions for BOLD Signal in Regions-of-Interest (ROI) to Food Cue Contrasts

eTable 10. Sex by Drink (Sucralose vs Sucrose) Interactions for BOLD Signal in Regions-of-Interest (ROI) to Food Cue Contrasts

eTable 11. Post-Hoc Analysis for BOLD Signal to Food Cue Contrasts Following Sucralose vs Sucrose Ingestion, Stratified by Sex

eTable 12. Sex Status by Drink (Sucrose vs Water) Interactions for BOLD Signal in Regions-of-Interest (ROI) to Food Cue Contrasts

eTable 13. Post-Hoc Analysis for BOLD Signal to Food Cue Contrasts Following Sucrose vs Water Ingestion, Stratified by Sex

eTable 14. Sex Status by Drink (Sucralose vs Water) Interactions for BOLD Signal in Regions-of-Interest (ROI) to Food Cue Contrasts

© 2021 Yunker AG et al. *JAMA Network Open.*

eTable 15. Post-Hoc Analysis for BOLD Signal to Food Cue Contrasts Following Sucralose vs Water Ingestion, Stratified by Sex

eTable 16. Post-Hoc Analysis for Differences in LSmeans Between Drink Comparisons for In-Scanner Cue-Induced Hunger, Wanting, and Liking Ratings in Whole Cohort

eTable 17. Post-Hoc Analysis for Differences in LSmeans Between Drink Comparisons for In-Scanner Cue-Induced Hunger Ratings, Stratified by BMI Status

eTable 18. Post-Hoc Analysis for Differences in LSmeans Between Drink Comparisons for In-Scanner Cue-Induced Hunger, Liking, and Wanting Ratings, Stratified by Sex

eTable 19. Post-Hoc 3-Way Interaction Between BMI Status, Sex, and Drink Condition (Sucralose vs Sucrose) on BOLD Signal in Regions-of-Interest (ROI) to Food Cue Contrasts

eFigure 1. Sagittal, Coronal, and Axial Images Depicting the Anatomically Defined Brain Regions of Interest (ROIs)

eFigure 2. Trajectories for Plasma Glucose and Hormones After Sucralose, Sucrose, and Water Drinks, Among Whole Cohort

eFigure 3. MFC BOLD Signal Response to Savory vs Non-Food Cues After Sucralose Compared to Sucrose Ingestion, Stratified by Both BMI Status and Sex

eFigure 4. Trajectories for Plasma Glucose and Hormones After Sucralose, Sucrose, and Water Drinks, Stratified by Body Mass Index (BMI) Status

eFigure 5. Trajectories for Plasma Glucose and Hormones After Sucralose, Sucrose, and Water Drinks, Stratified by Sex

eFigure 6. Difference in Total Caloric Intake Following Sucralose vs Sucrose Preload in the Whole Cohort and Stratified by Males and Females

eReferences

This supplemental material has been provided by the authors to give readers additional information about their work.

© 2021 Yunker AG et al. *JAMA Network Open.*

eAppendix 1. Supplemental Methods

Detailed Study Overview

All the visits were conducted in the morning after a 12h overnight fast. During the screening visit, we assessed eligibility for participation in the study and demographic information and anthropometric measurements were obtained. Height was measured to the nearest 0.1cm using a stadiometer and weight to the nearest 0.1kg using a bioelectrical impedance analysis scale (Model no. SC-331S, TANITA Corporation of American, Inc.), and BMI was calculated as weight in kilograms divided by height in meters squared. Participants were split into three BMI status groups (healthy weight: 18.5-24.9 kg/m^2, overweight: 25-29.9 kg/m^2, and obesity: ≥ 30 kg/m^2) based on Centers for Disease Control and Prevention (CDC) criteria [2]. Measurement of body weight was repeated at the beginning of each MRI study session. Participants with a weight change of ≥5 lbs between study sessions were disqualified to reduce variability in brain responses due to changes in body weight. Participants ingested 300mL drinks containing either sucrose (75g), sucralose (sweetness matched), or a water control, mixed with 0.45g of non-sweetened zero calorie cherry flavoring (Kraft Foods Kool-Aid® Unsweetened Cherry Drink Mix) for palatability. The sucrose and sucralose drinks were individually sweetness matched during the initial screening visit using a blinded task where participants selected the concentration of sucralose (1.5 mM, 2 mM, or 3 mM) that best matched a 25% weight per volume concentration of sucrose. The order of the drinks was randomized using a computer-generated sequence. Participants and experimenters were blind to the drink provided during the study sessions. Of the 76 participants in the trial who received at least one drink allocation, 2 participants received neither the sucrose nor sucralose drink allocations (due to study drop-out) and therefore were excluded from this analysis, while 74 participants received at least one of the primary drink condition allocations (i.e., sucrose and/or sucralose) and were included. Of these 74 subjects, 72 participants received both primary drink allocations, while 2 participants received only one primary drink allocation prior to withdrawing from the study (see **Figure 2** for additional details).

© 2021 Yunker AG et al. *JAMA Network Open.*

MRI scan included a T1 structural scan (for anatomical registration) followed by participants exiting the scanner to consume the test drink within two minutes in order to reduce variability in timing of drink effects. After consuming the drink, participants re-entered the scanner and underwent a food cue task (described below) beginning at approximately 20min post-drink ingestion. Arterial spin labelling (ASL) sequences were also collected as a part of the larger study but are not included in the current analysis. During each study visit, blood samples were collected at baseline (0min), 10min, 35min, and 120min post-drink to measure plasma glucose, insulin, GLP-1, acyl-ghrelin, PYY, and leptin concentrations. The study ended with a food buffet (125min post-drink, described below). Female subjects underwent study visits during the follicular phase of their menstrual cycles to reduce cycle related variability in hunger and food cravings [3,4].

MRI Imaging Analysis Details

To analyze fMRI data, we used several tools from the Oxford University Centre for Functional MRI of the Brain Software Library (FMRIB). MRI data were processed using the fMRI Expert Analysis Tool (FEAT) version 6.00. Eight functional volumes (8TRs) acquired at the beginning of each MRI session were discarded to account for magnetic saturation effects. fMRI data were preprocessed using motion correction, high-pass filtering (100s), and spatial smoothing with a Gaussian kernel of full width at half-maximum=5mm. Functional data were first mapped to each participant's anatomical image and then registered into standard space [Montreal Neurological Institute (MNI)] using affine transformation with FMRIB's Linear Image Registration Tool to the avg152 T1 MNI template. Food and non-food blocks were added to the general linear model (GLM) after convolution with a canonical hemodynamic response function. Temporal derivatives and temporal filtering were added, increasing statistical sensitivity. Motion confounds were generated using the tool "fsl_motion_outliers" to be used as no-interest regressors in the GLM. For each participant, visual cue contrast maps were created on the first-level analysis. All ROI

were bilateral and anatomically defined using the Harvard-Oxford atlas found in FSL (which provides probability mapping of 21 subcortical brain structures), except the hypothalamus, which is not included in the atlas and was defined bilaterally as a 2-mm spherical ROI surrounding peak glucose-responsive voxels identified previously [7]. Percent BOLD signal change was extracted from each ROI and cue contrast for each participant to identify differences in relative brain activation to food cues vs non-food cues using FSL's FEATquery.

Ad-libitum Buffet Meal

The buffet meal consisted of 32 pre-measured food and drink items. Total energy available from the buffet meal was 4650 kcal (for a full list of foods at the buffet and the calorie content of each food cue see **eTable 2**). Caloric value per gram or fluid ounce of each item was calculated using the Nutritional Data System for Research (NDSR) software (described below). Participants were given 20 minutes to eat any quantity they desired and instructed not to leave the room with any items. After the participant exited, each buffet item was re-weighed. Total caloric intake during the buffet meal was calculated using the difference between the pre-meal and post-meal weight for each buffet item.

To give an index of the degree of compensation for the 300kcal sucrose preload during the ad libitum buffet meal, we calculated COMPX scores using the following equation: COMPX = ((calories consumed after water condition – calories consumed after sucrose condition) / (300)) x 100 [10]. A score of 100% would indicate that the caloric intake consumed in the buffet meal was exactly 300kcal more in the water control condition to compensate for the caloric difference between sucrose (300kcal) and water (0kcal) preloads, whereas over 100% represents over-compensation for the preload calories (i.e., consuming too much after the water preload and/or too little after the sucrose preload), and 1-99% indicates some degree of compensation (i.e., consuming more after the water preload and/or less after the sucrose preload but not enough to fully compensate for the difference in preload calories) [10].

Habitual NNS Dietary Intake Assessment and Analysis

Diet was assessed using the multipass 24-hour dietary recall, which is a validated method that provides detailed information on food and beverages consumed over the previous 24-hour period [11,12]. Each dietary interview was administered by a trained staff member, wherein volunteers were asked to recall all food and drinks items (including meals and snacks) that they ingested during the previous 24-hours. In order to account for potential daily variations in dietary intake, 24-hour recalls were captured on both weekdays and weekend days. After the dietary recalls were obtained, the data was analyzed using the Nutritional Data System for Research (NDSR) software v.2018, developed by the Nutrition Coordinating Center, University of Minnesota, Minneapolis, MN, USA [13]. Dietary recalls were also assessed for plausibility and quality using the method described by Jones et al. [14], and using this method, 359 dietary recalls were included in the analysis (an average of 5 per participant) and 5 recalls were excluded. The variables outputted from this software that were used in the analysis were the average daily intake of acesulfame potassium (aceK), sucralose, saccharin, aspartame, or any combination thereof, in milligrams (mg). To represent habitual NNS dietary intake, we used each subjects' mean values across all recalls obtained. Participants were classified as either NNS user if average daily intake of NNS was above 0 or NNS non-user if average daily NNS intake was equal to 0.

Statistical Analysis Details

We found AUC values to be right-skewed; for statistical analyses, AUC values for glucose, insulin, GLP-1, acyl-ghrelin, PYY, and leptin were cubic root transformed to better meet the assumptions of normality. In addition, the residuals for the buffet meal models were not normally distributed, and for statistical analyses, total caloric was square root transformed. Paired t-tests and analysis of variance were used to compare NNS dietary intake between sex and BMI status

© 2021 Yunker AG et al. *JAMA Network Open.*

groups, respectively. Baseline levels of plasma glucose, insulin, and GLP-1 between the sucralose, sucrose, and water drink condition visits were compared using analysis of variance.

eAppendix 2. Supplementary Results of Secondary Outcomes and Additional Post-Hoc Results

Neural BOLD Signal to Food Cues after *Sucrose vs Water (Control) and Sucralose vs Water (Control)* Drink

Whole Cohort after Sucrose vs Water (control) and Sucralose vs Water (control)

There were no significant BOLD signal differences in any ROI in response to any food cue contrasts after either the sucrose vs water or sucralose vs water drink comparisons, adjusted for covariates (age, sex, BMI status, and NNS user status) and multiple ROI and visual cue contrasts (**eTables 4,5**).

Effects of BMI Status after Sucrose vs Water (control)

We observed BMI status x drink interactions in the MFC in response to high-calorie food vs non-food (p=0.02) and savory food vs non-food (p=0.04), adjusted for covariates (age, sex, and NNS user status), multiple ROIs, and visual cue contrast comparisons. The remaining associations did not meet the threshold of significance (**eTable 7**).

As a post-hoc analysis, we stratified results by BMI status (and adjusted for age, sex, and NNS user status as covariates) in order to understand the directionality of the interactions. However, in data stratified by BMI status, the associations did not meet the threshold of significance (**eTable 8**).

Effects of BMI Status after Sucralose vs Water (control)

None of the BMI status x drink interactions met the threshold of statistical significance (**eTable 9**).

Effects of Sex after Sucrose vs Water (control)

There were sex x drink interactions in response to food vs non-food cues in the MFC (p=0.03), hippocampus (p=0.03), and OFC (p=0.03), in response to high-calorie vs low-calorie food cues in the dorsal striatum (p=0.04), MFC (p=0.04), insula (p=0.04), and OFC (p=0.04), in response to sweet vs non-food cues in the MFC (p=0.02) and OFC (p=0.04), and after low-calorie vs non-food in the MFC (p=0.01), OFC (p=0.01), hippocampus (p=0.02), dorsal striatum (p=0.03), and insula (p=0.03), adjusted for covariates (age, BMI status, and NNS user status) and multiple ROIs and visual cue contrast comparisons. The remaining associations did not meet the threshold of significance (**eTable 12**).

As a post-hoc analysis, we stratified results by sex (and adjusted for age, BMI status, and NNS user status as covariates) to better understand the directionality of those interactions. MFC response to food vs non-food cues was greater after sucrose vs water ingestion among males (β=0.22, 95% CI: 0.03-0.40, p=0.02), whereas conversely, females had a decreased BOLD signal after sucrose vs water (β=-0.18, 95% CI: -0.33 to -0.02, p=0.02). Furthermore, after food vs non-food cues, males also had greater BOLD signal after sucrose vs water in the hippocampus (β=0.21, 95% CI: 0.07-0.34, p<0.01) and OFC (β=0.18, 95% CI: 0.02-0.35, p=0.03), whereas females did not have differences in reactivity between the sucrose and water conditions in those regions (**eTable 13**). After high-calorie vs low-calorie food cues, males had decreased dorsal striatum reactivity to sucrose relative to water ingestion (β=-0.14, 95% CI: -0.25 to -0.04, p=0.01), whereas females did not have differences in dorsal striatum reactivity between those drink conditions (**eTable 13**). MFC response to sweet vs non-food cues was greater after sucrose vs water ingestion among males (β=0.25, 95% CI: 0.03-0.48, p=0.02), whereas females had a decreased BOLD signal after sucrose vs water (β=-0.28, 95% CI: -0.48 to -0.08, p=0.01). After sweet vs non-food cues, females also had decreased OFC reactivity to sucrose vs water ingestion (β=-0.16, 95% CI: -0.28 to -0.03, p=0.01), whereas males did not have differences in OFC reactivity between those drink conditions (**eTable 13**). After low-calorie vs non-food cues, males

had increased MFC and OFC reactivity (β=0.28, 95% CI: 0.06-0.51, p=0.01 and β=0.25, 95% CI: 0.06-0.44, p=0.01, respectively), while females had decreased MFC and OFC reactivity (β=-0.20, 95% CI: -0.37 to -0.02, p=0.03 and β=-0.14, 95% CI: -0.25 to -0.04, p=0.01, respectively), after sucrose relative to water ingestion. Correspondingly, after low-calorie vs non-food cues, males had greater hippocampal (β=0.23, 95% CI: 0.05-0.40, p=0.01), dorsal striatum (β=0.16, 95% CI: 0.03-0.29, p=0.02), and insula (β=0.20, 95% CI: 0.03-0.36, p=0.02) reactivity after sucrose compared to water, while females did not have differential responses in those regions are the sucrose and water conditions (**eTable 13**).

Effects of Sex after Sucralose vs Water (control)

There were sex x drink interactions in response to food vs non-food cues in the MFC (p=0.03), hippocampus (p=0.03), and OFC (p=0.03), in response to high-calorie vs low-calorie food cues in the dorsal striatum (p=0.04), MFC (p=0.04), insula (p=0.04), and OFC (p=0.04), in response to sweet vs non-food cues in the MFC (p=0.02) and OFC (p=0.04), and after low-calorie vs non-food in the MFC (p=0.01), OFC (p=0.01), hippocampus (p=0.02), dorsal striatum (p=0.03), and insula (p=0.03), adjusted for covariates (age, BMI status, and NNS user status) and multiple ROIs and visual cue contrast comparisons. The remaining associations did not meet the threshold of significance (**eTable 14**).

As a post-hoc analysis, we stratified results by sex (and adjusted for age, BMI status, and NNS user status as covariates) to better understand the directionality of those interactions. Males had greater MFC and hippocampal BOLD signal to food vs non-food cues after sucralose vs water (β=0.24, 95% CI: 0.06-0.42, p=0.01 and β=0.15, 95% CI: 0.02-0.28, p=0.03, respectively), while females did not have differential MFC or hippocampal responses to those drink conditions (**eTable 15**). After high-calorie vs low-calorie cues, females had greater reactivity to sucralose vs water in the MFC (β=0.27, 95% CI: 0.10-0.43, p<0.01), OFC (β=0.12, 95% CI: 0.03-0.21, p=0.01), and insula (β=0.11, 95% CI: 0.02-0.20, p=0.02), while males did not have differential BOLD signal to

© 2021 Yunker AG et al. *JAMA Network Open.*

high-calorie vs low-calorie cues in those ROI (**eTable 15**). Males, but not females, had decreased BOLD signal in the dorsal striatum to high-calorie vs low-calorie cues after sucralose compared to water ingestion (β=-0.16, 95% CI: -0.26 to -0.05, p<0.01) (**eTable 15**). In contrast, males had increased hippocampal and MFC reactivity to low-calorie vs non-food cues (β=0.19, 95% CI: 0.02-0.36, p=0.03 and β=0.29, 95% CI: 0.07-0.52, p=0.01, respectively), while females had decreased MFC BOLD signal (β=-0.20, 95% CI: -0.38 to -0.03, p=0.03), after sucralose relative to water consumption (**eTable 15**). None of the remaining associations met the threshold of significance (**eTable 15**).

In-Scanner Food Cue-Induced Appetite Ratings after *Sucralose vs Sucrose (and vs Water control)* Drink

Whole Cohort

There was a main effect of drink on in-scanner cue-induced ratings of hunger after viewing food [F(2,139)=6.47, p<0.01], high-calorie [F(2,138)=5.32, p=0.01], low-calorie [F(2,138)=7.81, p<0.001], savory [F(2,139)=3.57, p=0.03], sweet [F(2,139)=5.57, p=0.01] and non-food [F(2,141)=7.59, p<0.01] blocks. We also observed a main effect of drink on ratings of wanting after viewing food [F(2,140)=4.09, p=0.02], high-calorie [F(2,140)=5.38, p=0.01], low-calorie [F(2,138)=7.41, p<0.01], and savory [F(2,142)=4.07, p=0.02] blocks, but not after sweet [F(2,140)=1.84, p=0.16] or non-food [F(2,142)=0.60, p=0.55] blocks. While we did observe a main effect of drink on ratings of liking after viewing savory foods [F(2,141)=5.14, p=0.01], there were no main effects of drink on ratings of liking after viewing food [F(2,140)=2.48, p=0.09], high-calorie [F(2,140)=2.37, p=0.10], low-calorie [F(2,137)=1.12, p=0.33], sweet [F(2,139)=0.33, p=0.72] or non-food [F(2,141)=1.38, p=0.25] blocks.

As a post-hoc analysis, we compared the difference of LSmeans ± SE between each drink comparison (i.e., sucralose vs sucrose, sucralose vs water, sucrose vs water) in order to

understand the directionality of each significant main effect of drink on in-scanner cue-induced appetite ratings (shown in **eTable 16**). We observed a main effect of drink on in-scanner cue-induced ratings of hunger and wanting, but not liking, across most food cues whereby hunger ratings after viewing food (β=0.25, 95% CI: 0.03-0.47, p=0.03), high-calorie (β=0.23, 95% CI: 0.01-0.45, p=0.04), low-calorie (β=0.28, 95% CI: 0.08-0.48, p=0.01), sweet (β=0.26, 95% CI: 0.02-0.50, p=0.03), and non-food (β=0.23, 95% CI: 0.03-0.43, p=0.03) cues were higher following sucralose vs sucrose (**eTable 16**). In secondary analyses, hunger and wanting ratings were also consistently higher after water vs sucrose conditions, and liking ratings after viewing savory food cues were higher following water compared to the sucralose and sucrose conditions (see **eTable 16** for details).

Effects of BMI Status

We observed BMI status x drink interactions on in-scanner cue-induced ratings of hunger after viewing low-calorie [F(4,135)=3.37, p=0.01], sweet [F(4,136)=3.15, p=0.02], and non-food blocks [F(4,137)=3.18, p=0.02], with similar trends after viewing food [F(4,135)=2.50, p=0.05] and high-calorie [F(4,135)=2.46, p=0.05] blocks, but not after the savory block [F(4,135)=1.39, p=0.24], adjusted for covariates (age, sex, and NNS user status). We did not observe BMI status by drink interactions for in-scanner cue-induced ratings of liking after viewing food [F(4,136)=0.42, p=0.80], high-calorie [F(4,136)=0.55, p=0.70], low-calorie [F(4,133)=2.01, p=0.10], savory [F(4,137)=0.37, p=0.83], sweet [F(4,135)=0.46, p=0.77], or non-food [F(4,137)=0.85, p=0.50] blocks, adjusted for covariates (age, sex, and NNS user status). Correspondingly, there were also no BMI status by drink interactions for in-scanner cue-induced ratings of wanting after viewing food [F(4,136)=0.31, p=0.87], high-calorie [F(4,136)=0.13, p=0.97], low-calorie [F(4,134)=0.93, p=0.45], savory [F(4,137)=0.90, p=0.47], sweet [F(4,136)=0.30, p=0.88], or non-food [F(4,138)=1.78, p=0.14] blocks, adjusted for covariates (age, sex, and NNS user status).

As a post-hoc analysis, we compared the difference of LSmeans ± SE between each drink comparison (i.e. sucralose vs sucrose, sucralose vs water, sucrose vs water) stratified by BMI status in order to understand the directionality of each significant BMI status x drink interaction on in-scanner cue-induced hunger ratings (shown in **eTable 17**). In analyses stratified by BMI group, individuals with overweight (β=0.52, 95% CI: 0.13-0.91, p=0.04), but not with healthy weight (β=0.36, 95% CI: 0.01-0.71, p=0.14) or obesity (β=-0.12, 95% CI: -0.57-0.33, p=0.88), had greater hunger after viewing sweet food cues following sucralose vs sucrose conditions. Secondary analyses showed that individuals with healthy weight and overweight, but not with obesity, reported greater hunger after water vs sucrose conditions across most food cue types, and participants with healthy weight also reported greater hunger to non-food cues following water vs sucralose (see **eTable 17** for details). There were no significant BMI status by drink interactions for in-scanner cue-induced ratings of liking or wanting (see **eAppendix 2** for details).

Effects of Sex

We observed sex x drink interactions on in-scanner cue-induced ratings of hunger after viewing food [F(2,136)=3.35, p=0.04] and sweet [F(2,136)=3.95, p=0.02] blocks, but not after viewing high-calorie [F(2,135)=1.93, p=0.15], low-calorie [F(2,136)=2.79, p=0.07], savory [F(2,136)=0.51, p=0.60], or non-food [F(2,139)=2.82, p=0.06] blocks, adjusted for covariates (age, BMI status, and NNS user status). We did not observe sex by drink interactions for in-scanner cue-induced ratings of liking after viewing food [F(2,138)=0.67, p=0.51], high-calorie [F(2,138)=0.71, p=0.49], low-calorie [F(2,135)=0.15, p=0.86], savory [F(2,139)=1.82, p=0.17], or non-food [F(2,139)=0.26, p=0.77] blocks, adjusted for covariates (age, BMI status, and NNS user status). There were also no sex by drink interactions for in-scanner cue-induced ratings of wanting after viewing food [F(2,138)=1.16, p=0.32], high-calorie [F(2,138)=1.81, p=0.17], low-calorie [F(2,136)=0.87, p=0.42], savory [F(2,139)=0.12, p=0.88], or non-food [F(2,140)=2.68, p=0.07] blocks, adjusted for covariates (age, BMI status, and NNS user status). However, we did observe significant sex x

© 2021 Yunker AG et al. *JAMA Network Open.*

drink interactions on in-scanner cue-induced ratings of both liking and wanting after viewing the sweet block [F(2,137)=3.60, p=0.03 and F(2,137)=3.21, p=0.04, respectively], adjusted for covariates (age, BMI status, and NNS user status).

As a post-hoc analysis, we compared the difference of LSmeans ± SE between each drink comparison (i.e. sucralose vs sucrose, sucralose vs water, sucrose vs water) stratified by sex in order to understand the directionality of each significant sex x drink interaction on in-scanner cue-induced hunger, liking, and wanting ratings (shown in **eTable 18**). Sex stratified analyses showed no differences in liking or wanting ratings after sweet food cues following sucralose vs sucrose among either females (β=0.08, 95% CI: -0.14-0.30, p=0.77 and β=0.28, 95% CI: 0.01-0.55, p=0.16, respectively) or males (β=-0.26, 95% CI: -0.51 to -0.01, p=0.16 and β=-0.15, 95% CI: -0.46-0.16, p=0.67, respectively), but secondary analyses showed that females, but not males, reported less wanting for sweet foods after sucrose vs water (see **eTable 18** for details).

Eating Behavior after *Sucrose vs Water (Control) and Sucralose vs Water (Control)* Drink Conditions

Whole Cohort

Mean degree of caloric compensation for the sucrose preload (i.e., adjustment in caloric intake based on caloric preload form sucrose drink) (COMPX) for the whole cohort was 34.84 ± 82.54%, with a range of -131% to 229%. Total caloric intake (mean, SD) was 886.38 ± 394.58 following sucralose, 825.11 ± 469.60 following sucrose, and 929.29 ± 400.46 following water ingestion.

Participants consumed fewer total calories (β=-2.00, 95% CI: -2.84 to -0.68, p<0.001) following sucrose vs water. There was no difference in ad libitum total caloric (β=-0.63, 95% CI: -1.62-0.52, p=0.25) intake after the sucralose vs water conditions.

© 2021 Yunker AG et al. *JAMA Network Open.*

Effects of BMI Status

Mean COMPX for individuals with healthy weight was 28.80 ± 89.54% with a range of -131% to 229%, with overweight was 42.85 ± 68.73% with a range of -40% to 222%, and with obesity was 32.87 ± 91.24% with a range of -121% to 219%. Total caloric intake (mean, SD) after each drink condition is as follows: healthy weight (sucralose: 908.18 ± 543.27, sucrose: 921.96 ± 627.26, and water: 990.45 ± 495.04), overweight (sucralose: 839.87 ± 229.65, sucrose: 629.90 ± 230.34, and water: 821.45 ± 263.07), and obesity (sucralose: 911.36 ± 336.96, sucrose: 856.30 ± 429.88, and water: 971.89 ± 388.12).

Effects of Sex

Mean COMPX for males was 3.55 ± 73.24% with a range of -130% to 122%, while mean COMPX for females was 58.30 ± 82.14% with a range of -109% to 229%. Total caloric intake (mean, SD) after each drink condition is as follows: males (sucralose: 1089.19 ± 436.77, sucrose: 1067.60 ± 563.60, and water: 1072.87 ± 461.51) and females (sucralose: 733.04 ± 278.01, sucrose: 647.68 ± 281.51, and water: 817.23 ± 306.98).

Following the sucralose compared to the water drink, females consumed fewer total calories (β=-1.48, 95% CI: -2.86 to -0.09, p=0.04) whereas total caloric intake did not differ in males β=0.35, 95% CI: -1.33 to 2.03, p=0.68). Females also had less total caloric intake after the sucrose vs water drinks (β=-3.21, 95% CI: -4.59 to -1.83, p<0.001) whereas caloric intake did not differ between these conditions in males (β=-0.34, 95% CI: -2.04-1.37, p=0.69).

Post-Hoc Results Stratified by BMI status and Sex on Neural BOLD Signal Response to Food vs Non-Food Cues after *Sucralose vs Sucrose* Drink

MFC BOLD response to savory vs non-food cues in females with obesity: Lsmeans [95% CI] for MFC BOLD signal after sucralose: 0.47 [95% CI, 0.22-0.72]; after sucrose: -0.26 [95% CI, -0.50

© 2021 Yunker AG et al. *JAMA Network Open.*

to -0.03]; p<0.001); males with obesity: (sucralose: 0.43 [95% CI, 0.13-0.72]; sucrose: 0.07 [95% CI, -0.24-0.39]; p=0.12); females with overweight (sucralose: 0.03 [95% CI, -0.24-0.29]; sucrose: 0.11 [95% CI, -0.16-0.37]; p=0.68); males with overweight (sucralose: 0.35 [95% CI, 0.15-0.56]; sucrose: 0.27 [95% CI, 0.07-0.47]; p=0.54); females with healthy weight (sucralose: -0.12 [95% CI, -0.32-0.08]; sucrose: 0.06 [95% CI, -0.14-0.25]; p=0.18); males with healthy weight (sucralose: 0.23 [95% CI, -0.01-0.46]; sucrose: 0.30 [95% CI, 0.06-0.53]; p=0.55).

© 2021 Yunker AG et al. *JAMA Network Open.*

eTable 1. Full List of Visual Cues and Respective Categories Used During the BOLD Food Cue Task

Visual Cue	Block Categories
Burger	Food/High-Calorie/Savory
French fries	Food/High-Calorie/Savory
Fried chicken	Food/High-Calorie/Savory
Mac & cheese	Food/High-Calorie/Savory
Pizza	Food/High-Calorie/Savory
Potato chips	Food/High-Calorie/Savory
Taco	Food/High-Calorie/Savory
Brownie	Food/High-Calorie/Sweet
Chocolate bar	Food/High-Calorie/Sweet
Chocolate chip cookie	Food/High-Calorie/Sweet
Cinnamon roll	Food/High-Calorie/Sweet
Cupcake	Food/High-Calorie/Sweet
Donut	Food/High-Calorie/Sweet
Gummies	Food/High-Calorie/Sweet
Ice cream	Food/High-Calorie/Sweet
M&Ms	Food/High-Calorie/Sweet
Apple	Food/Low-Calorie
Banana	Food/Low-Calorie
Bell pepper	Food/Low-Calorie
Blueberry	Food/Low-Calorie
Broccoli	Food/Low-Calorie
Carrots	Food/Low-Calorie
Chicken salad	Food/Low-Calorie
Cucumber	Food/Low-Calorie
Fruit salad	Food/Low-Calorie
Grape	Food/Low-Calorie
Hummus	Food/Low-Calorie
Mixed fruit	Food/Low-Calorie
Orange	Food/Low-Calorie
Popcorn	Food/Low-Calorie
Salad	Food/Low-Calorie
Salmon	Food/Low-Calorie
Basket	Non-Food
Bike	Non-Food
Book	Non-Food
Box	Non-Food
Bucket	Non-Food
Bus	Non-Food
Chair	Non-Food
Dock	Non-Food
Door	Non-Food
Field	Non-Food
Flower	Non-Food
Forest	Non-Food
Road	Non-Food
Staircase	Non-Food
Stapler	Non-Food
Waterfall	Non-Food

© 2021 Yunker AG et al. *JAMA Network Open.*

eTable 2. Foods and Drinks Available at the Ad Libitum Buffet Meal Along With the Caloric Content of Each. Total energy available from the buffet meal was 4650kcal.

Food	Caloric content (kcal/gram)
Lay's Potato Chips (Original) (1 bag)	5.46
Doritos (Nacho Cheese) (1 bag)	4.96
Sun Chips (Garden Salsa) (1 bag)	4.68
Cheetos Crunchy (1 bag)	5.67
Goldfish Crackers (1 bag)	4.74
Skinny Pop Popcorn	5.01
Oreos (4 cookies)	4.72
Famous Amos Minis (1 bag)	5.07
Pop-Tart (Frosted Strawberry) (1 pack)	3.91
Ritz Bitz Crackers (1 bag)	5.22
Pretzels (1 bag)	3.95
Hummus (1)	1.74
M&Ms (Plain)	4.92
Skittles	3.84
Chewy Granola Bar (Chocolate) (1)	4.20
Chewy Granola Bar (Peanut Butter) (1)	4.10
Mini Bagel (Plain) (2)	2.79
Philadelphia Cream Cheese (2 pack)	3.21
Mixed Nuts	6.07
Raisins (2 boxes)	2.99
Apples Slices (1 bag)	0.52
Baby Carrots	0.41
String Cheese (1)	2.55
Greek Yogurt (Strawberry)	0.82
Yoplait Yogurt (Strawberry)	1.02
**Drinks**	**Caloric content (kcal/gram)**
Coca Cola	0.37
Diet Coca Cola	0.02
Sprite	0.40
Honest Tea (Honey)	0.33
Vitamin Water (Açai blueberry pomegranate)	0.21
Starbucks Frappuccino (Vanilla)	0.70
Water	0.00

© 2021 Yunker AG et al. *JAMA Network Open.*

eTable 3. BOLD Signal in Regions-of-Interest (ROI) to Food Cue Contrasts Following Sucralose vs Sucrose Ingestion in Whole Cohort.

Food Cue Contrast	Brain ROI	β estimate	SE	Lower CI	Upper CI	P	FDR P
**Food vs Non-Food**	**Amygdala**	0.01	0.04	-0.07	0.09	0.73	0.85
	**Dorsal Striatum**	-0.02	0.04	-0.10	0.06	0.54	0.85
	**Hippocampus**	-0.03	0.04	-0.11	0.05	0.50	0.85
	**Hypothalamus**	0.11	0.05	0.01	0.21	0.02*	0.26
	**Insula**	-0.03	0.04	-0.11	0.05	0.42	0.85
	**MFC**	0.09	0.06	-0.03	0.21	0.13	0.81
	**Nucleus Accumbens**	0.04	0.05	-0.06	0.14	0.42	0.85
	**OFC**	0.02	0.04	-0.06	0.10	0.61	0.85
**High-Calorie vs Low-Calorie**	**Amygdala**	0.03	0.04	-0.05	0.11	0.42	0.85
	**Dorsal Striatum**	0.02	0.03	-0.04	0.08	0.59	0.85
	**Hippocampus**	0.03	0.03	-0.03	0.09	0.32	0.85
	**Hypothalamus**	-0.01	0.05	-0.11	0.09	0.87	0.94
	**Insula**	0.05	0.04	-0.03	0.13	0.23	0.85
	**MFC**	0.13	0.05	0.03	0.23	0.02*	0.26
	**Nucleus Accumbens**	0.03	0.05	-0.07	0.13	0.61	0.85
	**OFC**	0.03	0.04	-0.05	0.11	0.46	0.85
**High-Calorie vs Non-Food**	**Amygdala**	0.02	0.04	-0.06	0.10	0.59	0.85
	**Dorsal Striatum**	-0.01	0.04	-0.09	0.07	0.67	0.85
	**Hippocampus**	-0.01	0.04	-0.09	0.07	0.71	0.85
	**Hypothalamus**	0.10	0.05	0.00	0.20	0.05	0.37
	**Insula**	-0.01	0.04	-0.09	0.07	0.69	0.85
	**MFC**	0.12	0.06	0.00	0.24	0.03*	0.32
	**Nucleus Accumbens**	0.05	0.05	-0.05	0.15	0.37	0.85
	**OFC**	0.03	0.04	-0.05	0.11	0.50	0.85
**Low-Calorie vs Non-Food**	**Amygdala**	-0.01	0.04	-0.09	0.07	0.82	0.91
	**Dorsal Striatum**	-0.03	0.04	-0.11	0.05	0.39	0.85
	**Hippocampus**	-0.03	0.04	-0.11	0.05	0.51	0.85
	**Hypothalamus**	0.11	0.05	0.01	0.21	0.02*	0.26
	**Insula**	-0.06	0.04	-0.14	0.02	0.19	0.85
	**MFC**	0.00	0.06	-0.12	0.12	0.94	0.96
	**Nucleus Accumbens**	0.02	0.05	-0.08	0.12	0.71	0.85
	**OFC**	0.00	0.04	-0.08	0.08	0.96	0.96
**Savory vs Non-Food**	**Amygdala**	0.03	0.05	-0.07	0.13	0.55	0.85
	**Dorsal Striatum**	0.01	0.04	-0.07	0.09	0.89	0.95
	**Hippocampus**	0.01	0.04	-0.07	0.09	0.73	0.85
	**Hypothalamus**	0.07	0.06	-0.05	0.19	0.28	0.85
	**Insula**	0.00	0.04	-0.08	0.08	0.94	0.96
	**MFC**	0.14	0.07	0.00	0.28	0.06	0.39
	**Nucleus Accumbens**	0.07	0.06	-0.05	0.19	0.22	0.85
	**OFC**	0.03	0.05	-0.07	0.13	0.56	0.85
**Sweet vs Non-Food**	**Amygdala**	0.02	0.06	-0.10	0.14	0.73	0.85
	**Dorsal Striatum**	-0.03	0.05	-0.13	0.07	0.49	0.85
	**Hippocampus**	-0.04	0.05	-0.14	0.06	0.45	0.85
	**Hypothalamus**	0.13	0.06	0.01	0.25	0.02*	0.26
	**Insula**	-0.02	0.05	-0.12	0.08	0.66	0.85
	**MFC**	0.11	0.07	-0.03	0.25	0.16	0.84
	**Nucleus Accumbens**	0.02	0.07	-0.12	0.16	0.76	0.87
	**OFC**	0.03	0.06	-0.09	0.15	0.61	0.85

*indicates p values are statistically significant at p<0.05. P values adjusted for covariates (age, sex, BMI status, and NNS user status). FDR p values adjusted for 8 ROI and 6 food cue contrasts comparisons and covariates (age, sex, BMI status, and NNS user status).

© 2021 Yunker AG et al. *JAMA Network Open.*

eTable 4. BOLD Signal in Regions-of-Interest (ROI) to Food Cue Contrasts Following Sucrose vs Water Ingestion in Whole Cohort.

Food Cue Contrast	Brain ROI	β estimate	SE	Lower CI	Upper CI	P	FDR P
**Food vs Non-Food**	**Amygdala**	0.02	0.04	-0.06	0.10	0.68	0.85
	**Dorsal Striatum**	-0.01	0.04	-0.08	0.07	0.85	0.85
	**Hippocampus**	0.07	0.04	-0.01	0.14	0.10	0.77
	**Hypothalamus**	-0.06	0.05	-0.16	0.04	0.23	0.85
	**Insula**	0.02	0.04	-0.07	0.10	0.72	0.85
	**MFC**	-0.01	0.06	-0.14	0.11	0.82	0.85
	**Nucleus Accumbens**	-0.02	0.06	-0.14	0.09	0.70	0.85
	**OFC**	0.01	0.05	-0.09	0.12	0.78	0.85
**High-Calorie vs Low-Calorie**	**Amygdala**	-0.06	0.04	-0.14	0.02	0.15	0.59
	**Dorsal Striatum**	-0.04	0.03	-0.11	0.02	0.21	0.59
	**Hippocampus**	-0.04	0.04	-0.12	0.04	0.37	0.59
	**Hypothalamus**	-0.01	0.05	-0.11	0.09	0.79	0.90
	**Insula**	-0.04	0.05	-0.13	0.05	0.36	0.59
	**MFC**	-0.02	0.07	-0.15	0.11	0.76	0.90
	**Nucleus Accumbens**	-0.06	0.06	-0.18	0.05	0.27	0.59
	**OFC**	-0.01	0.05	-0.10	0.08	0.91	0.91
**High-Calorie vs Non-Food**	**Amygdala**	0.00	0.04	-0.08	0.07	0.90	1.00
	**Dorsal Striatum**	-0.02	0.04	-0.09	0.05	0.56	1.00
	**Hippocampus**	0.05	0.04	-0.02	0.12	0.18	0.98
	**Hypothalamus**	-0.06	0.05	-0.16	0.04	0.25	0.98
	**Insula**	0.00	0.04	-0.09	0.09	1.00	1.00
	**MFC**	-0.02	0.06	-0.14	0.10	0.73	1.00
	**Nucleus Accumbens**	-0.04	0.06	-0.16	0.07	0.46	1.00
	**OFC**	0.01	0.05	-0.09	0.11	0.82	1.00
**Low-Calorie vs Non-Food**	**Amygdala**	0.06	0.05	-0.14	0.05	0.23	0.79
	**Dorsal Striatum**	0.02	0.04	-0.12	0.07	0.59	0.86
	**Hippocampus**	0.07	0.05	-0.07	0.13	0.15	0.79
	**Hypothalamus**	-0.04	0.05	-0.16	0.08	0.39	0.79
	**Insula**	0.04	0.05	-0.11	0.08	0.39	0.79
	**MFC**	0.00	0.08	-0.21	0.09	1.00	1.00
	**Nucleus Accumbens**	0.02	0.06	-0.17	0.13	0.71	0.86
	**OFC**	0.02	0.06	-0.14	0.11	0.75	0.86
**Savory vs Non-Food**	**Amygdala**	0.04	0.05	-0.04	0.15	0.40	0.67
	**Dorsal Striatum**	-0.01	0.05	-0.06	0.11	0.75	0.78
	**Hippocampus**	0.07	0.04	-0.03	0.17	0.08	0.61
	**Hypothalamus**	-0.07	0.06	-0.14	0.06	0.23	0.67
	**Insula**	0.02	0.05	-0.06	0.14	0.68	0.78
	**MFC**	0.02	0.07	-0.15	0.15	0.78	0.78
	**Nucleus Accumbens**	-0.06	0.06	-0.10	0.15	0.38	0.67
	**OFC**	0.04	0.05	-0.09	0.13	0.42	0.67
**Sweet vs Non-Food**	**Amygdala**	-0.04	0.05	-0.06	0.14	0.37	0.80
	**Dorsal Striatum**	-0.03	0.05	-0.10	0.08	0.58	0.80
	**Hippocampus**	0.03	0.05	-0.01	0.15	0.56	0.80
	**Hypothalamus**	-0.04	0.06	-0.19	0.05	0.52	0.80
	**Insula**	-0.02	0.05	-0.08	0.12	0.75	0.80
	**MFC**	-0.06	0.08	-0.11	0.15	0.46	0.80
	**Nucleus Accumbens**	-0.02	0.08	-0.19	0.07	0.77	0.80
	**OFC**	-0.02	0.06	-0.06	0.14	0.80	0.80

*indicates p values are statistically significant at p<0.05. P values adjusted for covariates (age, sex, BMI status, and NNS user status). FDR p values adjusted for multiple ROI and food cue contrasts comparisons and covariates (age, sex, BMI status, and NNS user status).

© 2021 Yunker AG et al. *JAMA Network Open.*

eTable 5. BOLD Signal in Regions-of-Interest (ROI) to Food Cue Contrasts Following Sucralose vs Water Ingestion in Whole Cohort.

Food Cue Contrast	Brain ROI	β estimate	SE	Lower CI	Upper CI	P	FDR P
**Food vs Non-Food**	**Amygdala**	0.03	0.04	-0.06	0.12	0.49	0.76
	**Dorsal Striatum**	-0.02	0.04	-0.09	0.06	0.66	0.76
	**Hippocampus**	0.05	0.04	-0.03	0.13	0.21	0.60
	**Hypothalamus**	0.06	0.05	-0.05	0.16	0.30	0.60
	**Insula Cortex**	0.00	0.04	-0.09	0.08	0.96	0.96
	**MFC**	0.08	0.06	-0.04	0.20	0.19	0.60
	**Nucleus Accumbens**	0.02	0.05	-0.07	0.12	0.65	0.76
	**OFC**	0.05	0.04	-0.04	0.14	0.29	0.60
**High-Calorie vs Low Calorie**	**Amygdala**	-0.03	0.04	-0.12	0.06	0.48	0.77
	**Dorsal Striatum**	-0.04	0.04	-0.11	0.03	0.29	0.77
	**Hippocampus**	-0.01	0.04	-0.09	0.06	0.72	0.91
	**Hypothalamus**	-0.04	0.05	-0.13	0.06	0.43	0.77
	**Insula Cortex**	-0.01	0.05	-0.10	0.09	0.91	0.91
	**MFC**	0.10	0.07	-0.04	0.25	0.16	0.77
	**Nucleus Accumbens**	-0.04	0.05	-0.15	0.06	0.41	0.77
	**OFC**	0.01	0.05	-0.08	0.11	0.80	0.91
**High-Calorie vs Non-Food**	**Amygdala**	0.02	0.04	-0.07	0.11	0.69	0.92
	**Dorsal Striatum**	-0.03	0.04	-0.10	0.04	0.45	0.76
	**Hippocampus**	0.04	0.04	-0.04	0.12	0.29	0.76
	**Hypothalamus**	0.04	0.06	-0.07	0.15	0.48	0.76
	**Insula Cortex**	0.00	0.04	-0.09	0.08	0.93	0.93
	**MFC**	0.11	0.06	-0.01	0.22	0.06	0.51
	**Nucleus Accumbens**	0.01	0.05	-0.09	0.10	0.89	0.93
	**OFC**	0.05	0.04	-0.03	0.13	0.24	0.76
**Low-Calorie vs Non-Food**	**Amygdala**	0.05	0.05	-0.13	0.08	0.31	0.69
	**Dorsal Striatum**	0.01	0.04	-0.13	0.04	0.77	0.95
	**Hippocampus**	0.05	0.04	-0.09	0.09	0.22	0.69
	**Hypothalamus**	0.08	0.05	-0.04	0.22	0.15	0.69
	**Insula Cortex**	0.00	0.05	-0.13	0.08	0.95	0.95
	**MFC**	0.01	0.08	-0.10	0.20	0.94	0.95
	**Nucleus Accumbens**	0.05	0.05	-0.13	0.12	0.34	0.69
	**OFC**	0.04	0.05	-0.08	0.13	0.50	0.80
**Savory vs Non-Food**	**Amygdala**	0.06	0.05	-0.05	0.14	0.21	0.41
	**Dorsal Striatum**	0.00	0.04	-0.07	0.09	0.92	0.92
	**Hippocampus**	0.09	0.04	-0.03	0.14	0.05	0.19
	**Hypothalamus**	-0.01	0.07	-0.03	0.19	0.86	0.92
	**Insula Cortex**	0.02	0.05	-0.09	0.10	0.75	0.92
	**MFC**	0.17	0.07	-0.15	0.16	0.02	0.15
	**Nucleus Accumbens**	0.02	0.06	-0.05	0.16	0.80	0.92
	**OFC**	0.07	0.05	-0.07	0.14	0.17	0.41
**Sweet vs Non-Food**	**Amygdala**	-0.02	0.05	-0.04	0.16	0.65	0.90
	**Dorsal Striatum**	-0.05	0.04	-0.09	0.08	0.27	0.90
	**Hippocampus**	0.00	0.05	0.00	0.18	0.94	0.99
	**Hypothalamus**	0.09	0.06	-0.14	0.12	0.16	0.90
	**Insula Cortex**	-0.02	0.05	-0.08	0.12	0.68	0.90
	**MFC**	0.05	0.08	0.03	0.30	0.52	0.90
	**Nucleus Accumbens**	0.00	0.06	-0.10	0.13	0.99	0.99
	**OFC**	0.03	0.05	-0.03	0.17	0.61	0.90

*indicates p values are statistically significant at p<0.05. P values adjusted for covariates (age, sex, BMI status, and NNS user status). FDR p values adjusted for multiple ROI and food cue contrasts comparisons and covariates (age, sex, BMI status, and NNS user status).

© 2021 Yunker AG et al. *JAMA Network Open.*

eTable 6. BMI Status by Drink (Sucralose Vs Sucrose) Interactions for BOLD Signal in Regions-of-Interest (ROI) to Food Cue Contrasts.

Food Cue Contrast	Brain ROI	P	FDR P
**Food vs Non-Food**	**Amygdala**	0.62	0.71
	**Dorsal Striatum**	0.12	0.31
	**Hippocampus**	0.20	0.33
	**Hypothalamus**	0.50	0.67
	**Insula**	0.17	0.33
	**MFC**	0.02*	0.14
	**Nucleus Accumbens**	0.87	0.87
	**OFC**	0.05	0.21
**High-Calorie vs Low-Calorie**	**Amygdala**	0.94	0.94
	**Dorsal Striatum**	0.16	0.93
	**Hippocampus**	0.63	0.93
	**Hypothalamus**	0.64	0.93
	**Insula**	0.61	0.93
	**MFC**	0.85	0.94
	**Nucleus Accumbens**	0.50	0.93
	**OFC**	0.70	0.93
**High-Calorie vs Non-Food**	**Amygdala**	0.75	0.75
	**Dorsal Striatum**	0.06	0.17
	**Hippocampus**	0.16	0.26
	**Hypothalamus**	0.44	0.59
	**Insula**	0.11	0.21
	**MFC**	0.02*	0.15
	**Nucleus Accumbens**	0.70	0.75
	**OFC**	0.06	0.17
**Low-Calorie vs Non-Food**	**Amygdala**	0.44	0.68
	**Dorsal Striatum**	0.38	0.68
	**Hippocampus**	0.27	0.68
	**Hypothalamus**	0.86	0.90
	**Insula**	0.51	0.68
	**MFC**	0.11	0.68
	**Nucleus Accumbens**	0.90	0.90
	**OFC**	0.18	0.68
**Savory vs Non-Food**	**Amygdala**	0.10	0.16
	**Dorsal Striatum**	0.02*	0.05
	**Hippocampus**	0.02*	0.05
	**Hypothalamus**	0.24	0.28
	**Insula**	0.13	0.17
	**MFC**	<0.001*	**<0.001***
	**Nucleus Accumbens**	0.33	0.33
	**OFC**	<0.01*	**0.01***
**Sweet vs Non-Food**	**Amygdala**	0.55	0.91
	**Dorsal Striatum**	0.40	0.91
	**Hippocampus**	0.64	0.91
	**Hypothalamus**	0.80	0.91
	**Insula**	0.39	0.91
	**MFC**	0.98	0.98
	**Nucleus Accumbens**	0.70	0.91
	**OFC**	0.71	0.91

*indicates p values are statistically significant at $p<0.05$. P values adjusted for covariates (age, sex, BMI status, and NNS user status). FDR p values adjusted for multiple ROI and food cue contrasts comparisons and covariates (age, sex, and NNS user status).

© 2021 Yunker AG et al. *JAMA Network Open.*

eTable 7. BMI Status by Drink (Sucrose vs Water) Interactions for BOLD Signal in Regions-of-Interest (ROI) to Food Cue Contrasts.

Food Cue Contrast	Brain ROI	P	FDR P
**Food vs Non-Food**	**Amygdala**	0.47	0.91
	**Dorsal Striatum**	0.82	0.97
	**Hippocampus**	0.08	0.32
	**Hypothalamus**	0.47	0.91
	**Insula**	0.97	0.97
	**MFC**	0.01*	0.11
	**Nucleus Accumbens**	0.92	0.97
	**OFC**	0.57	0.91
**High-Calorie vs Low-Calorie**	**Amygdala**	0.76	0.92
	**Dorsal Striatum**	0.64	0.92
	**Hippocampus**	0.37	0.75
	**Hypothalamus**	0.92	0.92
	**Insula**	0.85	0.92
	**MFC**	0.16	0.75
	**Nucleus Accumbens**	0.32	0.75
	**OFC**	0.37	0.75
**High-Calorie vs Non-Food**	**Amygdala**	0.43	0.79
	**Dorsal Striatum**	0.71	0.92
	**Hippocampus**	0.13	0.53
	**Hypothalamus**	0.49	0.79
	**Insula**	0.92	0.92
	**MFC**	<0.01*	**0.02***
	**Nucleus Accumbens**	0.81	0.92
	**OFC**	0.41	0.79
**Low-Calorie vs Non-Food**	**Amygdala**	0.65	0.98
	**Dorsal Striatum**	0.98	0.98
	**Hippocampus**	0.05	0.36
	**Hypothalamus**	0.59	0.98
	**Insula**	0.94	0.98
	**MFC**	0.27	0.98
	**Nucleus Accumbens**	0.53	0.98
	**OFC**	0.98	0.98
**Savory vs Non-Food**	**Amygdala**	0.67	0.77
	**Dorsal Striatum**	0.91	0.91
	**Hippocampus**	0.47	0.77
	**Hypothalamus**	0.60	0.77
	**Insula**	0.59	0.77
	**MFC**	<0.01*	**0.04***
	**Nucleus Accumbens**	0.64	0.77
	**OFC**	0.41	0.77
**Sweet vs Non-Food**	**Amygdala**	0.38	0.63
	**Dorsal Striatum**	0.63	0.63
	**Hippocampus**	0.12	0.47
	**Hypothalamus**	0.60	0.63
	**Insula**	0.43	0.63
	**MFC**	0.03*	0.26
	**Nucleus Accumbens**	0.42	0.63
	**OFC**	0.53	0.63

*indicates p values are statistically significant at p<0.05. P values adjusted for covariates (age, sex, BMI status, and NNS user status). FDR p values adjusted for multiple ROI and food cue contrasts comparisons and covariates (age, sex, and NNS user status).

© 2021 Yunker AG et al. *JAMA Network Open.*

eTable 8. Post-Hoc Analysis for MFC BOLD Signal to High-Calorie vs Non-Food and Savory vs Non-Food Contrasts Following Sucrose vs Water Ingestion, Stratified by BMI Status.

Food Cue Contrast[a]	Brain ROI[a]	BMI Group	β estimate	SE	Lower CI	Upper CI	P
**High-Calorie vs Non-Food**	**MFC**	Healthy Weight	0.10	0.08	-0.08	0.27	0.26
		Overweight	0.00	0.10	-0.20	0.21	0.97
		Obesity	-0.19	0.11	-0.43	0.05	0.11
**Savory vs Non-Food**		Healthy Weight	0.17	0.10	-0.04	0.37	0.10
		Overweight	0.02	0.12	-0.21	0.26	0.84
		Obesity	-0.16	0.11	-0.39	0.06	0.15

[a]Post-hoc analysis stratified by BMI status for ROI/food cue contrasts with a significant BMI status x drink interaction.

*indicates p values are statistically significant at p<0.05. Adjusted for covariates (age, BMI status, and NNS user status).

eTable 9. BMI Status by Drink (Sucralose vs Water) Interactions for BOLD Signal in Regions-of-Interest (ROI) to Food Cue Contrasts.

Food Cue Contrasts	Brain ROI	P	FDR P
**Food vs Non-Food**	**Amygdala**	0.67	0.90
	**Dorsal Striatum**	0.07	0.57
	**Hippocampus**	0.78	0.90
	**Hypothalamus**	0.34	0.68
	**Insula**	0.31	0.68
	**MFC**	1.00	1.00
	**Nucleus Accumbens**	0.74	0.90
	**OFC**	0.19	0.68
**High-Calorie vs Low-Calorie**	**Amygdala**	0.88	0.90
	**Dorsal Striatum**	0.22	0.60
	**Hippocampus**	0.20	0.60
	**Hypothalamus**	0.11	0.60
	**Insula**	0.53	0.80
	**MFC**	0.46	0.80
	**Nucleus Accumbens**	0.60	0.80
	**OFC**	0.90	0.90
**High-Calorie vs Non-Food**	**Amygdala**	0.73	0.87
	**Dorsal Striatum**	0.04*	0.29
	**Hippocampus**	0.52	0.83
	**Hypothalamus**	0.18	0.50
	**Insula**	0.25	0.50
	**MFC**	0.76	0.87
	**Nucleus Accumbens**	0.92	0.92
	**OFC**	0.25	0.50
**Low-Calorie vs Non-Food**	**Amygdala**	0.65	0.78
	**Dorsal Striatum**	0.45	0.78
	**Hippocampus**	0.63	0.78
	**Hypothalamus**	0.95	0.95
	**Insula**	0.68	0.78
	**MFC**	0.66	0.78
	**Nucleus Accumbens**	0.55	0.78
	**OFC**	0.26	0.78
**Savory vs Non-Food**	**Amygdala**	0.13	0.19
	**Dorsal Striatum**	0.04*	0.19
	**Hippocampus**	0.14	0.19
	**Hypothalamus**	0.10	0.19
	**Insula**	0.16	0.19
	**MFC**	0.19	0.19
	**Nucleus Accumbens**	0.19	0.19
	**OFC**	0.11	0.19
**Sweet vs Non-Food**	**Amygdala**	0.40	0.73
	**Dorsal Striatum**	0.20	0.73
	**Hippocampus**	0.70	0.73
	**Hypothalamus**	0.57	0.73
	**Insula**	0.59	0.73
	**MFC**	0.10	0.73
	**Nucleus Accumbens**	0.28	0.73
	**OFC**	0.73	0.73

*indicates p values are statistically significant at p<0.05. P values adjusted for covariates (age, sex, BMI status, and NNS user status). FDR p values adjusted for multiple ROI and food cue contrasts comparisons and covariates (age, sex, and NNS user status).

© 2021 Yunker AG et al. *JAMA Network Open.*

eTable 10. Sex by Drink (Sucralose vs Sucrose) Interactions for BOLD Signal in Regions-of-Interest (ROI) to Food Cue Contrasts.

Food Cue Contrast	Brain ROI	P	FDR P
**Food vs Non-Food**	**Amygdala**	0.25	0.33
	**Dorsal Striatum**	0.15	0.31
	**Hippocampus**	0.01*	**0.03***
	**Hypothalamus**	0.68	0.68
	**Insula**	0.22	0.33
	**MFC**	0.01*	**0.03***
	**Nucleus Accumbens**	0.60	0.68
	**OFC**	0.01*	**0.03***
**High-Calorie vs Low-Calorie**	**Amygdala**	0.35	0.40
	**Dorsal Striatum**	0.01*	**0.04***
	**Hippocampus**	0.21	0.28
	**Hypothalamus**	0.57	0.57
	**Insula**	0.01*	**0.04***
	**MFC**	0.01*	**0.04***
	**Nucleus Accumbens**	0.18	0.28
	**OFC**	0.02*	**0.04***
**High-Calorie vs Non-Food**	**Amygdala**	0.54	0.57
	**Dorsal Striatum**	0.22	0.44
	**Hippocampus**	0.05	0.15
	**Hypothalamus**	0.57	0.57
	**Insula**	0.27	0.44
	**MFC**	0.05	0.15
	**Nucleus Accumbens**	0.36	0.48
	**OFC**	0.04*	0.15
**Low-Calorie vs Non-Food**	**Amygdala**	0.07	0.09
	**Dorsal Striatum**	0.02*	**0.03***
	**Hippocampus**	0.01*	**0.02***
	**Hypothalamus**	0.90	0.90
	**Insula**	0.02*	**0.03***
	**MFC**	<0.01*	**0.01***
	**Nucleus Accumbens**	0.68	0.78
	**OFC**	<0.01*	**0.01***
**Savory vs Non-Food**	**Amygdala**	0.39	0.62
	**Dorsal Striatum**	0.11	0.44
	**Hippocampus**	0.61	0.70
	**Hypothalamus**	0.59	0.70
	**Insula**	0.07	0.44
	**MFC**	0.84	0.84
	**Nucleus Accumbens**	0.37	0.62
	**OFC**	0.37	0.62
**Sweet vs Non-Food**	**Amygdala**	0.05	0.10
	**Dorsal Striatum**	0.45	0.55
	**Hippocampus**	0.02*	0.05
	**Hypothalamus**	0.50	0.55
	**Insula**	0.55	0.55
	**MFC**	<0.01*	**0.02***
	**Nucleus Accumbens**	0.51	0.55
	**OFC**	0.01*	**0.04***

*indicates p values are statistically significant at p<0.05. P values adjusted for covariates (age, sex, BMI status, and NNS user status). FDR p values adjusted for multiple ROI and food cue contrasts comparisons and covariates (age, BMI status, and NNS user status).

© 2021 Yunker AG et al. *JAMA Network Open.*

eTable 11. Post-Hoc Analysis for BOLD Signal to Food Cue Contrasts Following Sucralose vs Sucrose Ingestion, Stratified by Sex.

Food Cue Contrast[a]	Brain ROI[a]	Sex	β estimate	SE	Lower CI	Upper CI	P
**Food vs Non-Food**	**Hippocampus**	male	-0.06	0.07	-0.20	0.08	0.40
		female	0.01	0.04	-0.07	0.09	0.83
	**MFC**	male	0.02	0.09	-0.16	0.20	0.80
		female	0.14	0.08	-0.02	0.30	0.07
	**OFC**	male	-0.08	0.08	-0.24	0.08	0.32
		female	0.12	0.05	0.02	0.22	**0.03***
**High-Calorie vs Low-Calorie**	**Dorsal Striatum**	male	-0.01	0.05	-0.11	0.09	0.82
		female	0.02	0.04	-0.06	0.10	0.61
	**Insula**	male	-0.01	0.08	-0.17	0.15	0.88
		female	0.07	0.05	-0.03	0.17	0.11
	**MFC**	male	0.01	0.10	-0.19	0.21	0.90
		female	0.21	0.08	0.05	0.37	**0.01***
	**OFC**	male	-0.01	0.08	-0.17	0.15	0.89
		female	0.04	0.05	-0.06	0.14	0.34
**Low-Calorie vs Non-Food**	**Dorsal Striatum**	male	-0.07	0.07	-0.21	0.07	0.30
		female	0.03	0.05	-0.07	0.13	0.56
	**Hippocampus**	male	-0.04	0.09	-0.22	0.14	0.68
		female	0.00	0.04	-0.08	0.08	0.91
	**Insula**	male	-0.07	0.08	-0.23	0.09	0.39
		female	-0.02	0.05	-0.12	0.08	0.73
	**MFC**	male	0.01	0.11	-0.21	0.23	0.91
		female	-0.01	0.09	-0.19	0.17	0.92
	**OFC**	male	-0.07	0.09	-0.25	0.11	0.47
		female	0.08	0.05	-0.02	0.18	0.14
**Sweet vs Non-Food**	**MFC**	male	-0.04	0.11	-0.26	0.18	0.69
		female	0.22	0.10	0.02	0.42	**0.03***
	**OFC**	male	-0.11	0.10	-0.31	0.09	0.31
		female	0.15	0.06	0.03	0.27	**0.01***

[a]Post-hoc analysis stratified by sex for ROI/food cue contrasts with a significant sex status x drink interaction.
*indicates p values are statistically significant at $p<0.05$. Adjusted for covariates (age, BMI status, and NNS user status).

© 2021 Yunker AG et al. *JAMA Network Open.*

eTable 12. Sex Status by Drink (Sucrose vs Water) Interactions for BOLD Signal in Regions-of-Interest (ROI) to Food Cue Contrasts.

Food Cue Contrast	Brain ROI	P	FDR P
**Food vs Non-Food**	**Amygdala**	0.25	0.33
	**Dorsal Striatum**	0.15	0.31
	**Hippocampus**	0.01*	**0.03***
	**Hypothalamus**	0.68	0.68
	**Insula**	0.22	0.33
	**MFC**	0.01*	**0.03***
	**Nucleus Accumbens**	0.60	0.68
	**OFC**	0.01*	**0.03***
**High-Calorie vs Low-Calorie**	**Amygdala**	0.35	0.40
	**Dorsal Striatum**	0.01*	**0.04***
	**Hippocampus**	0.21	0.28
	**Hypothalamus**	0.57	0.57
	**Insula**	0.01*	**0.04***
	**MFC**	0.01*	**0.04***
	**Nucleus Accumbens**	0.18	0.28
	**OFC**	0.02*	**0.04***
**High-Calorie vs Non-Food**	**Amygdala**	0.54	0.57
	**Dorsal Striatum**	0.22	0.44
	**Hippocampus**	0.05	0.15
	**Hypothalamus**	0.57	0.57
	**Insula**	0.27	0.44
	**MFC**	0.05	0.15
	**Nucleus Accumbens**	0.36	0.48
	**OFC**	0.04*	0.15
**Low-Calorie vs Non-Food**	**Amygdala**	0.07	0.09
	**Dorsal Striatum**	0.02*	**0.03***
	**Hippocampus**	0.01*	**0.02***
	**Hypothalamus**	0.90	0.90
	**Insula**	0.02*	**0.03***
	**MFC**	<0.01*	**0.01***
	**Nucleus Accumbens**	0.68	0.78
	**OFC**	<0.01*	**0.01***
**Savory vs Non-Food**	**Amygdala**	0.39	0.62
	**Dorsal Striatum**	0.11	0.44
	**Hippocampus**	0.61	0.70
	**Hypothalamus**	0.59	0.70
	**Insula**	0.07	0.44
	**MFC**	0.84	0.84
	**Nucleus Accumbens**	0.37	0.62
	**OFC**	0.37	0.62
**Sweet vs Non-Food**	**Amygdala**	0.05	0.10
	**Dorsal Striatum**	0.45	0.55
	**Hippocampus**	0.02*	0.05
	**Hypothalamus**	0.50	0.55
	**Insula**	0.55	0.55
	**MFC**	<0.01*	**0.02***
	**Nucleus Accumbens**	0.51	0.55
	**OFC**	0.01*	**0.04***

*indicates p values are statistically significant at p<0.05. P values adjusted for covariates (age, sex, BMI status, and NNS user status). FDR p values adjusted for multiple ROI and food cue contrasts comparisons and covariates (age, BMI status, and NNS user status).

© 2021 Yunker AG et al. *JAMA Network Open.*

eTable 13. Post-Hoc Analysis for BOLD Signal to Food Cue Contrasts Following Sucrose vs Water Ingestion, Stratified by Sex.

Food Cue Contrast[a]	Brain ROI[a]	Sex	β Estimate	SE	Lower CI	Upper CI	P
**Food vs Non-Food**	**Hippocampus**	male	0.21	0.07	0.07	0.34	**<0.01***
		female	-0.03	0.04	-0.12	0.05	0.46
	**MFC**	male	0.22	0.09	0.03	0.40	**0.02***
		female	-0.18	0.08	-0.33	-0.02	**0.02***
	**OFC**	male	0.18	0.08	0.02	0.35	**0.03***
		female	-0.10	0.05	-0.20	0.00	0.05
**High Calorie vs Low Calorie**	**Dorsal Striatum**	male	-0.14	0.05	-0.25	-0.04	**0.01***
		female	0.03	0.04	-0.05	0.11	0.49
	**Insula**	male	-0.15	0.08	-0.32	0.01	0.07
		female	0.03	0.05	-0.06	0.13	0.45
	**MFC**	male	-0.12	0.10	-0.31	0.07	0.20
		female	0.05	0.08	-0.11	0.22	0.54
	**OFC**	male	-0.12	0.08	-0.28	0.05	0.15
		female	0.07	0.05	-0.02	0.16	0.11
**Low Calorie vs Non-Food**	**Dorsal Striatum**	male	0.16	0.07	0.03	0.29	**0.02***
		female	-0.07	0.05	-0.16	0.02	0.12
	**Hippocampus**	male	0.23	0.09	0.05	0.40	**0.01***
		female	-0.04	0.04	-0.12	0.05	0.38
	**Insula**	male	0.20	0.08	0.03	0.36	**0.02***
		female	-0.06	0.05	-0.17	0.04	0.24
	**MFC**	male	0.28	0.11	0.06	0.51	**0.01***
		female	-0.20	0.09	-0.37	-0.02	**0.03***
	**OFC**	male	0.25	0.09	0.06	0.44	**0.01***
		female	-0.14	0.05	-0.25	-0.04	**0.01***
**Sweet vs Non-Food**	**MFC**	male	0.25	0.11	0.03	0.48	**0.02***
		female	-0.28	0.10	-0.48	-0.08	**0.01***
	**OFC**	male	0.19	0.10	-0.02	0.40	0.08
		female	-0.16	0.06	-0.28	-0.03	**0.01***

[a]Post-hoc analysis stratified by sex for ROI/food cue contrasts with a significant sex status x drink interaction.
*indicates p values are statistically significant at p<0.05. Adjusted for covariates (age, BMI status, and NNS user status).

© 2021 Yunker AG et al. *JAMA Network Open.*

eTable 14. Sex Status by Drink (Sucralose vs Water) Interactions for BOLD Signal in Regions-of-Interest (ROI) to Food Cue Contrasts.

Food Cue Contrast	Brain ROI	P	FDR P
**Food vs Non-Food**	**Amygdala**	0.25	0.33
	**Dorsal Striatum**	0.15	0.31
	**Hippocampus**	0.01*	**0.03***
	**Hypothalamus**	0.68	0.68
	**Insula**	0.22	0.33
	**MFC**	0.01*	**0.03***
	**Nucleus Accumbens**	0.60	0.68
	**OFC**	0.01*	**0.03***
**High-Calorie vs Low-Calorie**	**Amygdala**	0.35	0.40
	**Dorsal Striatum**	0.01*	**0.04***
	**Hippocampus**	0.21	0.28
	**Hypothalamus**	0.57	0.57
	**Insula**	0.01*	**0.04***
	**MFC**	0.01*	**0.04***
	**Nucleus Accumbens**	0.18	0.28
	**OFC**	0.02*	**0.04***
**High-Calorie vs Non-Food**	**Amygdala**	0.54	0.57
	**Dorsal Striatum**	0.22	0.44
	**Hippocampus**	0.05	0.15
	**Hypothalamus**	0.57	0.57
	**Insula**	0.27	0.44
	**MFC**	0.05	0.15
	**Nucleus Accumbens**	0.36	0.48
	**OFC**	0.04*	0.15
**Low-Calorie vs Non-Food**	**Amygdala**	0.07	0.09
	**Dorsal Striatum**	0.02*	**0.03***
	**Hippocampus**	0.01*	**0.02***
	**Hypothalamus**	0.90	0.90
	**Insula**	0.02*	**0.03***
	**MFC**	<0.01*	**0.01***
	**Nucleus Accumbens**	0.68	0.78
	**OFC**	<0.01*	**0.01***
**Savory vs Non-Food**	**Amygdala**	0.39	0.62
	**Dorsal Striatum**	0.11	0.44
	**Hippocampus**	0.61	0.70
	**Hypothalamus**	0.59	0.70
	**Insula**	0.07	0.44
	**MFC**	0.84	0.84
	**Nucleus Accumbens**	0.37	0.62
	**OFC**	0.37	0.62
**Sweet vs Non-Food**	**Amygdala**	0.05	0.10
	**Dorsal Striatum**	0.45	0.55
	**Hippocampus**	0.02*	0.05
	**Hypothalamus**	0.50	0.55
	**Insula**	0.55	0.55
	**MFC**	<0.01*	**0.02***
	**Nucleus Accumbens**	0.51	0.55
	**OFC**	0.01*	**0.04***

*indicates p values are statistically significant at p<0.05. P values adjusted for covariates (age, sex, BMI status, and NNS user status). FDR p values adjusted for multiple ROI and food cue contrasts comparisons and covariates (age, BMI status, and NNS user status).

© 2021 Yunker AG et al. *JAMA Network Open.*

eTable 15. Post-Hoc Analysis for BOLD Signal to Food Cue Contrasts Following Sucralose vs Water Ingestion, Stratified by Sex.

Food Cue Contrast[a]	Brain ROI[a]	Sex	β Estimate	SE	Lower CI	Upper CI	P
**Food vs Non-Food**	**Hippocampus**	male	0.15	0.07	0.02	0.28	**0.03***
		female	-0.02	0.04	-0.11	0.06	0.61
	**MFC**	male	0.24	0.09	0.06	0.42	**0.01***
		female	-0.03	0.08	-0.19	0.12	0.66
	**OFC**	male	0.10	0.08	-0.06	0.26	0.21
		female	0.01	0.05	-0.09	0.12	0.79
**High-Calorie vs Low-Calorie**	**Dorsal Striatum**	male	-0.16	0.05	-0.26	-0.05	**<0.01***
		female	0.05	0.04	-0.03	0.13	0.24
	**Insula**	male	-0.16	0.08	-0.33	0.00	0.05
		female	0.11	0.05	0.02	0.20	**0.02***
	**MFC**	male	-0.11	0.10	-0.30	0.08	0.25
		female	0.27	0.09	0.10	0.43	**<0.01***
	**OFC**	male	-0.13	0.08	-0.29	0.03	0.12
		female	0.12	0.05	0.03	0.21	**0.01***
**Low-Calorie vs Non-Food**	**Dorsal Striatum**	male	0.09	0.07	-0.04	0.22	0.16
		female	-0.05	0.05	-0.14	0.05	0.33
	**Hippocampus**	male	0.19	0.09	0.02	0.36	**0.03***
		female	-0.04	0.04	-0.13	0.04	0.33
	**Insula**	male	0.13	0.08	-0.04	0.29	0.13
		female	-0.08	0.05	-0.19	0.03	0.13
	**MFC**	male	0.29	0.11	0.07	0.52	**0.01***
		female	-0.20	0.09	-0.38	-0.03	**0.03***
	**OFC**	male	0.18	0.09	-0.01	0.37	0.06
		female	-0.06	0.05	-0.17	0.05	0.26
**Sweet vs Non-Food**	**MFC**	male	0.21	0.11	-0.01	0.43	0.06
		female	-0.06	0.10	-0.26	0.14	0.56
	**OFC**	male	0.08	0.10	-0.13	0.29	0.44
		female	0.00	0.06	-0.13	0.12	0.95

[a]Post-hoc analysis stratified by sex for ROI/food cue contrasts with a significant sex status x drink interaction.
*indicates p values are statistically significant at p<0.05. Adjusted for covariates (age, BMI status, and NNS user status).

© 2021 Yunker AG et al. *JAMA Network Open.*

eTable 16. Post-Hoc Analysis for Differences in LSmeans Between Drink Comparisons for In-Scanner Cue-Induced Hunger, Wanting, and Liking Ratings in Whole Cohort.

Hunger						
**Visual Cue Block[a]**	**Drink Comparison**	**β estimate**	**SE**	**Lower CI**	**Upper CI**	**P**
**Food**	Sucralose vs Sucrose	0.25	0.11	0.03	0.47	**0.03***
	Sucralose vs Water	-0.14	0.11	-0.36	0.08	0.20
	Sucrose vs Water	-0.39	0.11	-0.61	-0.17	**<0.01***
**High-Calorie**	Sucralose vs Sucrose	0.23	0.11	0.01	0.45	**0.04***
	Sucralose vs Water	-0.12	0.11	-0.34	0.10	0.27
	Sucrose vs Water	-0.35	0.11	-0.57	-0.13	**<0.01***
**Low-Calorie**	Sucralose vs Sucrose	0.28	0.10	0.08	0.48	**0.01***
	Sucralose vs Water	-0.13	0.11	-0.35	0.09	0.23
	Sucrose vs Water	-0.40	0.10	-0.60	-0.20	**<0.001***
**Savory**	Sucralose vs Sucrose	0.20	0.12	-0.04	0.44	0.10
	Sucralose vs Water	-0.11	0.12	-0.35	0.13	0.34
	Sucrose vs Water	-0.31	0.12	-0.55	-0.07	**0.01***
**Sweet**	Sucralose vs Sucrose	0.26	0.12	0.02	0.50	**0.03***
	Sucralose vs Water	-0.13	0.12	-0.37	0.11	0.28
	Sucrose vs Water	-0.39	0.12	-0.63	-0.15	**<0.01***
**Non-Food**	Sucralose vs Sucrose	0.23	0.10	0.03	0.43	**0.03***
	Sucralose vs Water	-0.18	0.11	-0.40	0.04	0.09
	Sucrose vs Water	-0.41	0.10	-0.61	-0.21	**<0.001***
**Wanting**						
**Visual Cue Block[a]**	**Drink Comparison**	**β estimate**	**SE**	**Lower CI**	**Upper CI**	**P**
**Food**	Sucralose vs Sucrose	0.10	0.10	-0.10	0.30	0.28
	Sucralose vs Water	-0.17	0.10	-0.37	0.03	0.08
	Sucrose vs Water	-0.27	0.10	-0.47	-0.07	**0.01***
**High-Calorie**	Sucralose vs Sucrose	0.12	0.08	-0.04	0.28	0.12
	Sucralose vs Water	-0.14	0.08	-0.30	0.02	0.09
	Sucrose vs Water	-0.26	0.08	-0.42	-0.10	**<0.01***
**Low-Calorie**	Sucralose vs Sucrose	0.18	0.08	0.02	0.34	**0.03***
	Sucralose vs Water	-0.13	0.08	-0.29	0.03	0.12
	Sucrose vs Water	-0.30	0.08	-0.46	-0.14	**<0.001***
**Savory**	Sucralose vs Sucrose	0.13	0.11	-0.09	0.35	0.25
	Sucralose vs Water	-0.19	0.11	-0.41	0.03	0.10
	Sucrose vs Water	-0.32	0.11	-0.54	-0.10	**0.01***
**Liking**						
**Visual Cue Block[a]**	**Drink Comparison**	**β estimate**	**SE**	**Lower CI**	**Upper CI**	**P**
**Savory**	Sucralose vs Sucrose	0.08	0.09	-0.10	0.26	0.40
	Sucralose vs Water	-0.21	0.09	-0.39	-0.03	**0.03***
	Sucrose vs Water	-0.28	0.09	-0.46	-0.10	**<0.01***

[a]Post-hoc analysis for appetite ratings and visual cue blocks with a significant main effect of drink.
*indicates p values are statistically significant at p<0.05. Adjusted for covariates (age, sex, BMI status, and NNS user status).

© 2021 Yunker AG et al. *JAMA Network Open.*

eTable 17. Post-Hoc Analysis for Differences in LSmeans Between Drink Comparisons for In-Scanner Cue-Induced Hunger Ratings, Stratified by BMI Status.

**Hunger: Healthy Weight**						
**Visual Cue Block[a]**	**Drink Comparison**	**β estimate**	**SE**	**Lower CI**	**Upper CI**	**P**
**Low-Calorie**	Sucralose vs Sucrose	0.40	0.18	0.05	0.75	0.08
	Sucralose vs Water	-0.38	0.18	-0.73	-0.03	0.11
	Sucrose vs Water	-0.78	0.18	-1.13	-0.43	**<0.001***
**Sweet**	Sucralose vs Sucrose	0.36	0.18	0.01	0.71	0.14
	Sucralose vs Water	-0.34	0.18	-0.69	0.01	0.18
	Sucrose vs Water	-0.70	0.18	-1.05	-0.35	**<0.01***
**Non-Food**	Sucralose vs Sucrose	0.37	0.15	0.08	0.66	0.07
	Sucralose vs Water	-0.43	0.16	-0.74	-0.12	**0.03***
	Sucrose vs Water	-0.79	0.15	-1.08	-0.50	**<0.001***
**Hunger: Overweight**						
**Visual Cue Block[a]**	**Drink Comparison**	**β estimate**	**SE**	**Lower CI**	**Upper CI**	**P**
**Low-Calorie**	Sucralose vs Sucrose	0.38	0.19	0.01	0.75	0.15
	Sucralose vs Water	-0.09	0.19	-0.46	0.28	0.89
	Sucrose vs Water	-0.47	0.19	-0.84	-0.10	0.06
**Sweet**	Sucralose vs Sucrose	0.52	0.20	0.13	0.91	**0.04***
	Sucralose vs Water	-0.06	0.20	-0.45	0.33	0.95
	Sucrose vs Water	-0.58	0.20	-0.97	-0.19	**0.02***
**Non-Food**	Sucralose vs Sucrose	0.32	0.19	-0.05	0.69	0.25
	Sucralose vs Water	-0.13	0.19	-0.50	0.24	0.80
	Sucrose vs Water	-0.45	0.19	-0.82	-0.08	0.07
**Hunger: Obesity**						
**Visual Cue Block[a]**	**Drink Comparison**	**β estimate**	**SE**	**Lower CI**	**Upper CI**	**P**
**Low-Calorie**	Sucralose vs Sucrose	0.03	0.16	-0.28	0.34	0.99
	Sucralose vs Water	0.13	0.16	-0.18	0.44	0.73
	Sucrose vs Water	0.10	0.16	-0.21	0.41	0.82
**Sweet**	Sucralose vs Sucrose	-0.12	0.23	-0.57	0.33	0.88
	Sucralose vs Water	0.07	0.23	-0.38	0.52	0.96
	Sucrose vs Water	0.19	0.23	-0.26	0.64	0.72
**Non-Food**	Sucralose vs Sucrose	-0.03	0.18	-0.38	0.32	0.99
	Sucralose vs Water	0.05	0.18	-0.30	0.40	0.96
	Sucrose vs Water	0.08	0.18	-0.27	0.43	0.91

[a]Post-hoc analysis stratified by BMI status for appetite ratings and visual cue blocks with significant BMI by drink interactions.
*indicates p values are statistically significant at p<0.05. Adjusted for covariates (age, sex, and NNS user status).

© 2021 Yunker AG et al. *JAMA Network Open.*

eTable 18. Post-Hoc Analysis for Differences in LSmeans Between Drink Comparisons for In-Scanner Cue-Induced Hunger, Liking, and Wanting Ratings, Stratified by Sex.

Hunger: Males						
**Visual Cue Block[a]**	**Drink Comparison**	**β estimate**	**SE**	**Lower CI**	**Upper CI**	**P**
**Food**	Sucralose vs Sucrose	-0.07	0.14	-0.34	0.20	0.88
	Sucralose vs Water	-0.31	0.14	-0.58	-0.04	0.09
	Sucrose vs Water	-0.24	0.14	-0.51	0.03	0.23
**Sweet**	Sucralose vs Sucrose	-0.11	0.15	-0.40	0.18	0.75
	Sucralose vs Water	-0.30	0.15	-0.59	-0.01	0.14
	Sucrose vs Water	-0.18	0.15	-0.47	0.11	0.46
**Hunger: Females**						
**Visual Cue Block[a]**	**Drink Comparison**	**β estimate**	**SE**	**Lower CI**	**Upper CI**	**P**
**Food**	Sucralose vs Sucrose	0.50	0.16	0.19	0.81	**0.01***
	Sucralose vs Water	0.00	0.16	-0.31	0.31	1.00
	Sucrose vs Water	-0.50	0.16	-0.81	-0.19	**0.01***
**Sweet**	Sucralose vs Sucrose	0.54	0.17	0.21	0.87	**0.01***
	Sucralose vs Water	0.00	0.17	-0.33	0.33	1.00
	Sucrose vs Water	-0.54	0.17	-0.87	-0.21	**0.01***
**Liking: Males**						
**Visual Cue Block[a]**	**Drink Comparison**	**β estimate**	**SE**	**Lower CI**	**Upper CI**	**P**
**Sweet**	Sucralose vs Sucrose	-0.26	0.13	-0.51	-0.01	0.16
	Sucralose vs Water	0.02	0.13	-0.23	0.27	0.98
	Sucrose vs Water	0.28	0.13	0.03	0.53	0.11
**Liking: Females**						
**Visual Cue Block[a]**	**Drink Comparison**	**β estimate**	**SE**	**Lower CI**	**Upper CI**	**P**
**Sweet**	Sucralose vs Sucrose	0.08	0.11	-0.14	0.30	0.77
	Sucralose vs Water	-0.08	0.11	-0.30	0.14	0.76
	Sucrose vs Water	-0.16	0.11	-0.38	0.06	0.34
**Wanting: Males**						
**Visual Cue Block[a]**	**Drink Comparison**	**β estimate**	**SE**	**Lower CI**	**Upper CI**	**P**
**Sweet**	Sucralose vs Sucrose	-0.15	0.16	-0.46	0.16	0.67
	Sucralose vs Water	-0.06	0.16	-0.37	0.25	0.95
	Sucrose vs Water	0.09	0.16	-0.22	0.40	0.86
**Wanting: Females**						
**Visual Cue Block[a]**	**Drink Comparison**	**β estimate**	**SE**	**Lower CI**	**Upper CI**	**P**
**Sweet**	Sucralose vs Sucrose	0.28	0.14	0.01	0.55	0.16
	Sucralose vs Water	-0.16	0.15	-0.45	0.13	0.56
	Sucrose vs Water	-0.44	0.15	-0.73	-0.15	**0.01***

[a]Post-hoc analysis stratified by sex for appetite ratings and visual cue blocks with significant sex by drink interactions.
*indicates p values are statistically significant at p<0.05. Adjusted for covariates (age, sex, and NNS user status).

© 2021 Yunker AG et al. *JAMA Network Open.*

eTable 19. Post-Hoc 3-Way Interaction Between BMI Status, Sex, and Drink Condition (Sucralose vs Sucrose) on BOLD Signal in Regions-of-Interest (ROI) to Food Cue Contrasts.

Food Cue Contrast	Brain ROI	P	FDR P
**Food vs Non-Food**	**Amygdala**	0.62	0.83
	**Dorsal Striatum**	0.14	0.38
	**Hippocampus**	0.09	0.35
	**Hypothalamus**	0.78	0.89
	**Insula**	0.44	0.71
	**MFC**	0.05	0.35
	**Nucleus Accumbens**	0.98	0.98
	**OFC**	0.19	0.38
**High-Calorie vs Low-Calorie**	**Amygdala**	0.68	0.68
	**Dorsal Striatum**	0.18	0.57
	**Hippocampus**	0.56	0.68
	**Hypothalamus**	0.40	0.63
	**Insula**	0.64	0.68
	**MFC**	0.19	0.57
	**Nucleus Accumbens**	0.25	0.57
	**OFC**	0.29	0.57
**High-Calorie vs Non-Food**	**Amygdala**	0.63	0.72
	**Dorsal Striatum**	0.05	0.21
	**Hippocampus**	0.09	0.23
	**Hypothalamus**	0.58	0.72
	**Insula**	0.32	0.51
	**MFC**	0.02*	0.15
	**Nucleus Accumbens**	0.78	0.78
	**OFC**	0.12	0.24
**Low-Calorie vs Non-Food**	**Amygdala**	0.59	0.97
	**Dorsal Striatum**	0.79	0.97
	**Hippocampus**	0.06	0.48
	**Hypothalamus**	0.97	0.97
	**Insula**	0.89	0.97
	**MFC**	0.29	0.97
	**Nucleus Accumbens**	0.73	0.97
	**OFC**	0.38	0.97
**Savory vs Non-Food**	**Amygdala**	0.17	0.27
	**Dorsal Striatum**	0.09	0.18
	**Hippocampus**	0.04*	0.15
	**Hypothalamus**	0.22	0.29
	**Insula**	0.40	0.46
	**MFC**	<0.01*	**0.02***
	**Nucleus Accumbens**	0.55	0.55
	**OFC**	0.08	0.18
**Sweet vs Non-Food**	**Amygdala**	0.50	0.57
	**Dorsal Striatum**	0.20	0.57
	**Hippocampus**	0.34	0.57
	**Hypothalamus**	0.90	0.90
	**Insula**	0.37	0.57
	**MFC**	0.14	0.57
	**Nucleus Accumbens**	0.31	0.57
	**OFC**	0.44	0.57

*indicates p values are statistically significant at p<0.05. P values adjusted for covariates (age, sex, BMI status, and NNS user status). FDR p values adjusted for multiple ROI and food cue contrasts comparisons and covariates (age, BMI status, and NNS user status).

© 2021 Yunker AG et al. *JAMA Network Open.*

eFigures

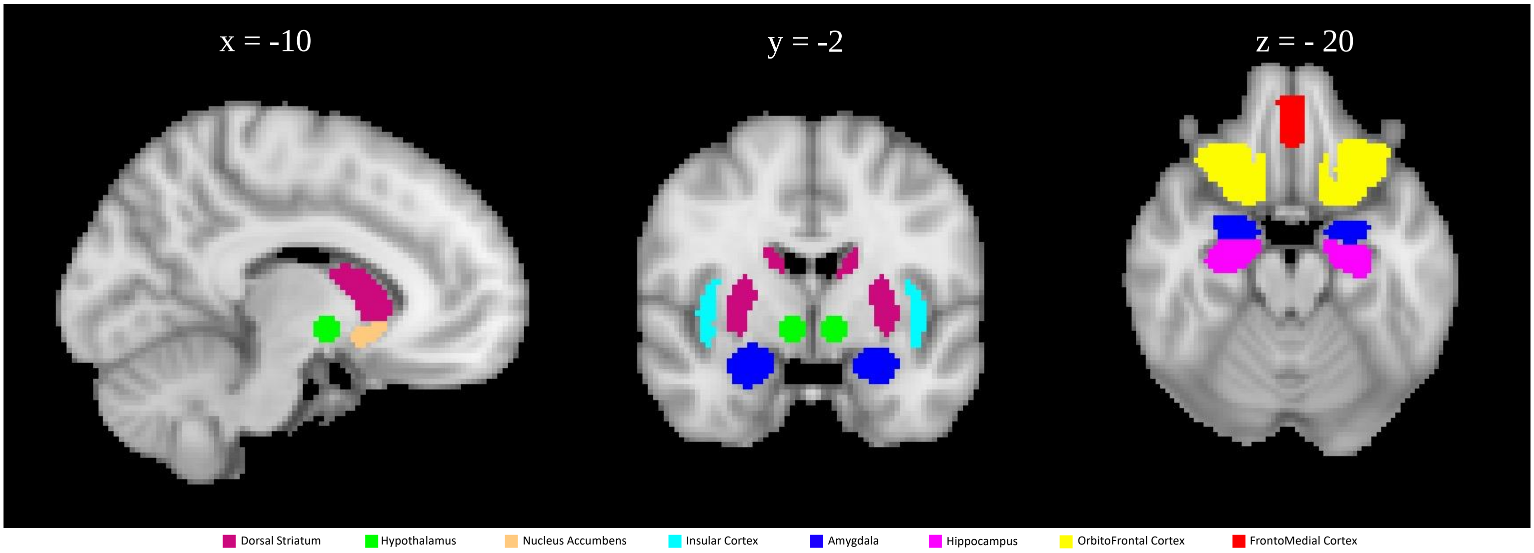

eFigure 1. Sagittal, Coronal, and Axial Images Depicting the Anatomically Defined Brain Regions of Interest (ROIs)

© 2021 Yunker AG et al. *JAMA Network Open.*

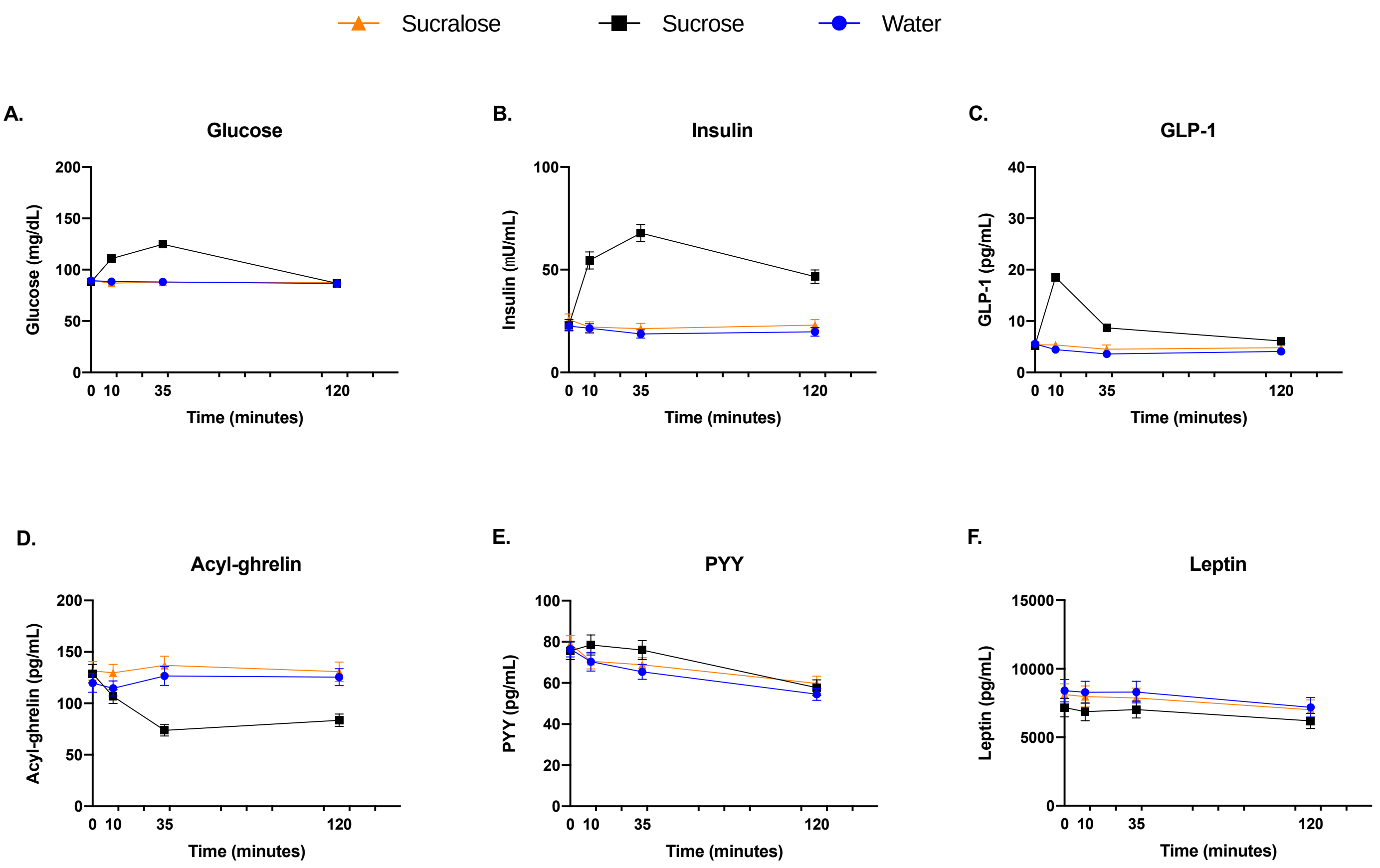

eFigure 2. Trajectories for Plasma Glucose and Hormones After Sucralose, Sucrose, and Water Drinks, Among Whole Cohort **A.** plasma glucose; **B.** insulin; and **C.** glucagon-like peptide-1 (GLP-1); **D.** acyl-ghrelin; **E.** peptide YY (PYY); and **F.** leptin following sucralose (orange), sucrose (black), and water (blue) drinks among whole cohort. Data in the figure are expressed as unadjusted mean ± SEM for visual/interpretive purposes, but all statistical analyses were based on AUC and adjusted for covariates (age, sex, BMI status, and NNS user status).

© 2021 Yunker AG et al. *JAMA Network Open.*

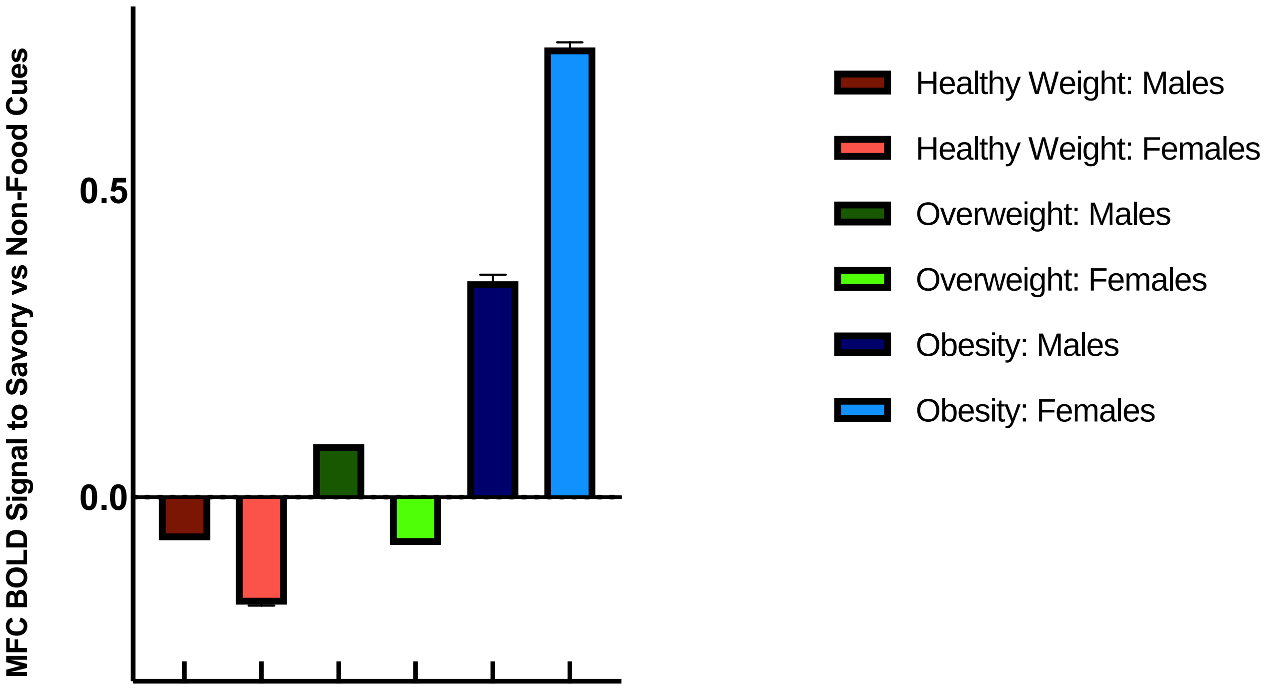

eFigure 3. MFC BOLD Signal Response to Savory vs Non-Food Cues After Sucralose Compared to Sucrose Ingestion, Stratified by Both BMI Status and Sex. Data expressed as LSmeans ± SE difference between sucralose compared to sucrose.

© 2021 Yunker AG et al. *JAMA Network Open.*

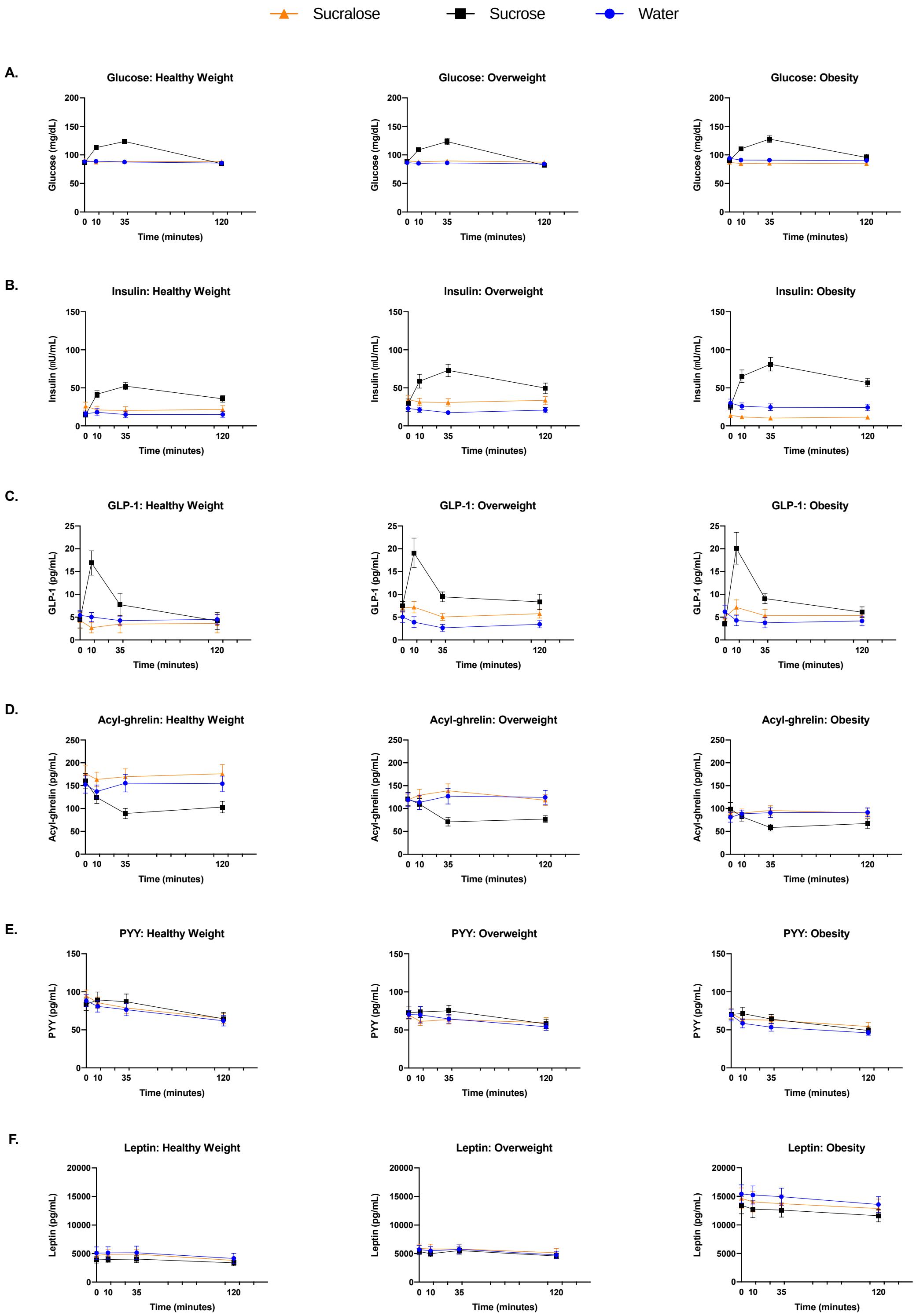

eFigure 4. Trajectories for Plasma Glucose and Hormones After Sucralose, Sucrose, and Water Drinks, Stratified by Body Mass Index (BMI) Status **A.** glucose; **B.** insulin; **C.** glucagon-like peptide-1 (GLP-1); **D.** acyl-ghrelin; **E.** peptide YY (PYY); and **F.** leptin values after sucralose (orange), sucrose (black), and water (blue) drinks, stratified by body mass index (BMI) status. Data are expressed as unadjusted ± SEM for visual/interpretive purposes, but all statistical analyses were based on AUC and adjusted for covariates (age, sex, and NNS user status).

© 2021 Yunker AG et al. *JAMA Network Open.*

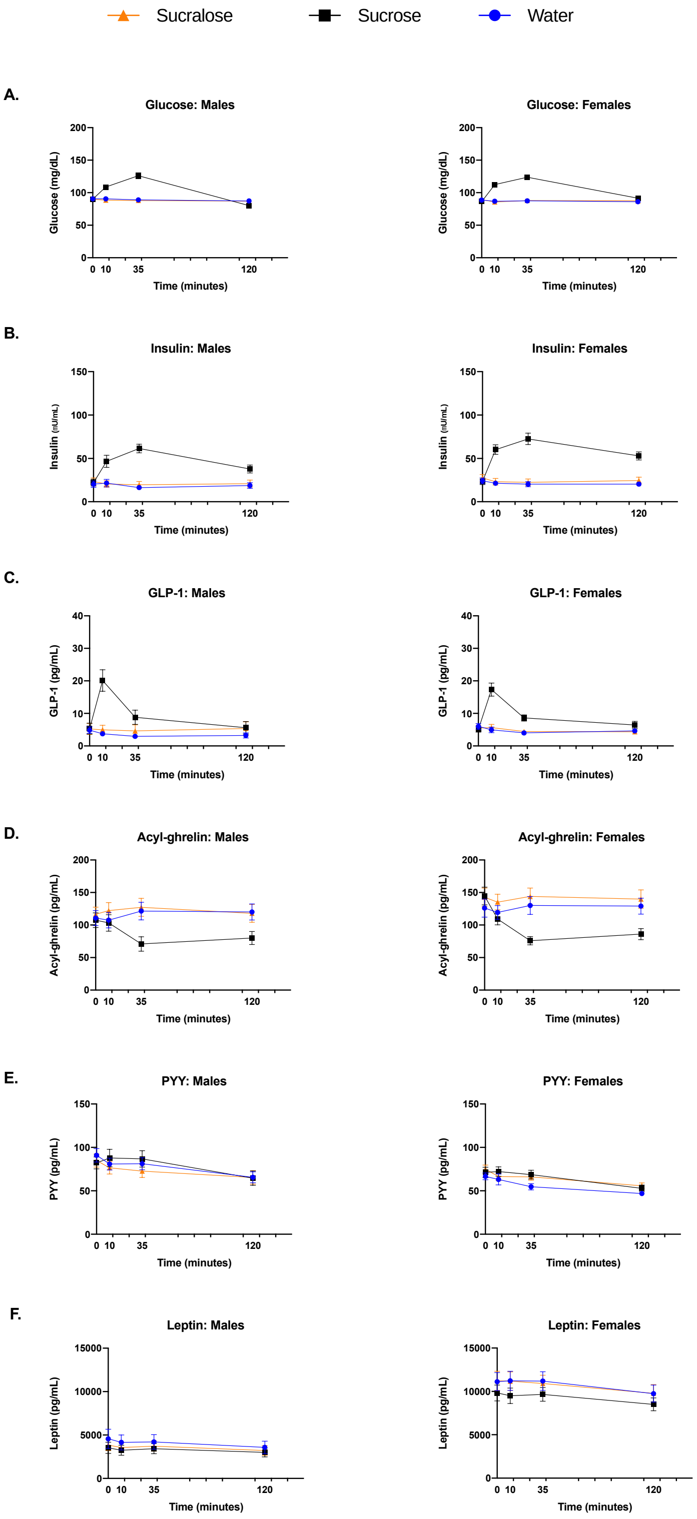

eFigure 5. Trajectories for Plasma Glucose and Hormones After Sucralose, Sucrose, and Water Drinks, Stratified by Sex **A.** glucose; **B.** insulin; **C.** glucagon-like peptide-1 (GLP-1); **D.** acyl-ghrelin; **E.** peptide YY (PYY); and **F.** leptin values after sucralose (orange), sucrose (black), and water (blue) drinks, stratified by sex. Data are expressed as unadjusted ± SEM for

© 2021 Yunker AG et al. *JAMA Network Open.*

visual/interpretive purposes, but all statistical analyses were based on AUC and adjusted for covariates (age, sex, and NNS user status).

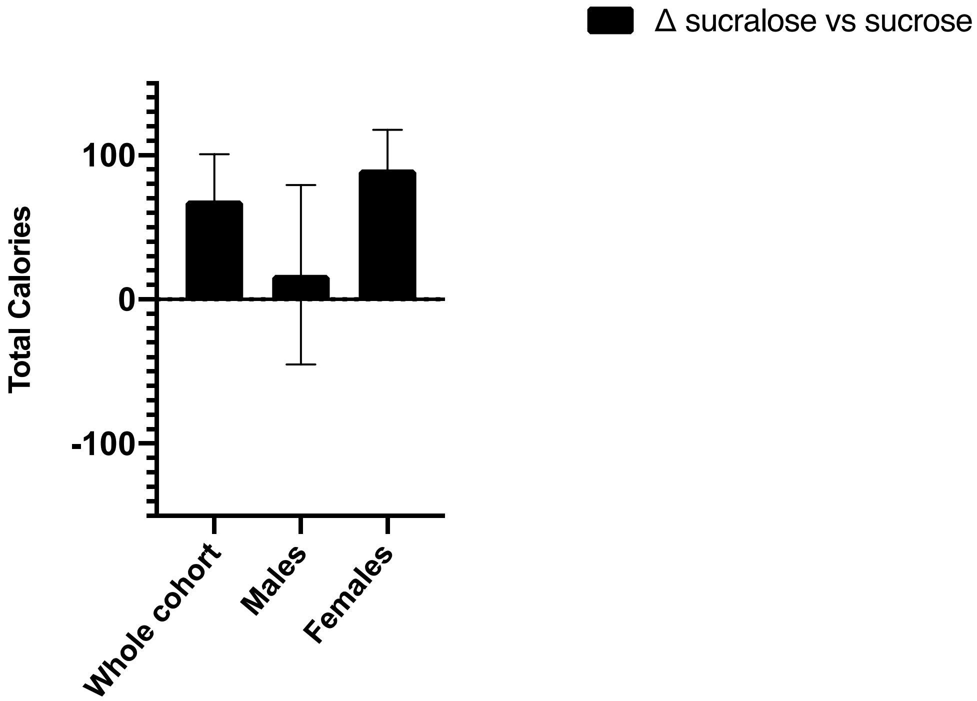

eFigure 6. Difference in Total Caloric Intake Following Sucralose vs Sucrose Preload in the Whole Cohort and Stratified by Males and Females. Data in the figure are expressed as unadjusted mean ± SEM for visual/interpretive purposes, but all statistical analyses were adjusted for covariates (age, BMI status, and NNS user status).

© 2021 Yunker AG et al. *JAMA Network Open.*

eReferences

1. Page K, Luo S, Dorton H, et al. Neural mechanisms for appetitive response for high reward foods. Published online July 30, 2020. doi:10.17605/OSF.IO/E7B9F

2. CDC. Defining Adult Overweight and Obesity | Overweight & Obesity. Accessed January 10, 2018. https://www.cdc.gov/obesity/adult/defining.html

3. Dye L, Blundell JE. Menstrual cycle and appetite control: implications for weight regulation. *Hum Reprod*. 1997;12(6):1142-1151. doi:10.1093/humrep/12.6.1142

4. Krishnan S, Tryon RR, Horn WF, Welch L, Keim NL. Estradiol, SHBG and leptin interplay with food craving and intake across the menstrual cycle. *Physiol Behav*. 2016;165:304-312. doi:10.1016/j.physbeh.2016.08.010

5. Tang DW, Fellows LK, Small DM, Dagher A. Food and drug cues activate similar brain regions: A meta-analysis of functional MRI studies. *Physiol Behav*. 2012;106(3):317-324. doi:10.1016/j.physbeh.2012.03.009

6. Chen J, Papies EK, Barsalou LW. A core eating network and its modulations underlie diverse eating phenomena. *Brain Cogn*. 2016;110:20-42. doi:10.1016/j.bandc.2016.04.004

7. Page KA, Seo D, Belfort-DeAguiar R, et al. Circulating glucose levels modulate neural control of desire for high-calorie foods in humans. *J Clin Invest*. 2011;121(10):4161-4169. doi:10.1172/JCI57873

8. Blechert J, Meule A, Busch NA, Ohla K. Food-pics: An image database for experimental research on eating and appetite. *Front Psychol*. 2014;5:617.

9. Dorton HM, Luo S, Monterosso JR, Page KA. Influences of Dietary Added Sugar Consumption on Striatal Food-Cue Reactivity and Postprandial GLP-1 Response. *Front Psychiatry*. 2018;8:297.

10. Carnell S, Benson L, Gibson EL, Mais LA, Warkentin S. Caloric compensation in preschool children: Relationships with body mass and differences by food category. *Appetite*. 2017;116:82-89. doi:10.1016/j.appet.2017.04.018

11. Johnson RK, Driscoll P, Goran MI. Comparison of Multiple-Pass 24-Hour Recall Estimates of Energy Intake With Total Energy Expenditure Determined By the Doubly Labeled Water Method in Young Children. *J Am Diet Assoc*. 1996;96(11):1140-1144. doi:10.1016/S0002-8223(96)00293-3

12. Biro B, Hulshof KFAM, Ovesen L, Cruz JA. Selection of methodology to assess food intake. *Eur J Clin Nutr*. 2002;56(S2):S25.

13. Feskanich D, Sielaff BH, Chong K, Buzzard IM. Computerized collection and analysis of dietary intake information. *Comput Methods Programs Biomed*. 1989;30(1):47-57. doi:10.1016/0169-2607(89)90122-3

© 2021 Yunker AG et al. *JAMA Network Open.*

14. Jones S, Luo S, Dorton HM, et al. Evidence of a Role for the Hippocampus in Food-Cue Processing and the Association with Body Weight and Dietary Added Sugar. *Obesity*. 2021;29(2):370-378. doi:https://doi.org/10.1002/oby.23085

© 2021 Yunker AG et al. *JAMA Network Open.*
