# Supplementary material for: Obesity and Sex-Related Associations With Differential Effects of Sucralose vs Sucrose on Appetite and Reward Processing: A Randomized Crossover Trial
Source: JAMA Netw Open. 2021 Sep 28;4(9):e2126313. doi: 10.1001/jamanetworkopen.2021.26313 (PMC8479585; doi:10.1001/jamanetworkopen.2021.26313)
Supplement: Supplement 3. — Data Sharing Statement [file jamanetwopen-e2126313-s003.pdf]

Data Sharing Statement

Yunker. Obesity and Sex-Related Associations With Differential Effects of Sucralose vs Sucrose on Appetite and Reward Processing. *JAMA Netw Open*. Published September 28, 2021. doi:10.1001/jamanetworkopen.2021.26313

Data
Data available: No

Additional Information
Explanation for why data not available: The datasets generated and analyzed during the current study are available from the corresponding author (K.A.P.) on reasonable request, and all brain imaging data are available in an online digital repository (see reference 30 in the manuscript).
